# Assessing Quality of Care of Elderly Patients Using the ACOVE Quality Indicator Set: A Systematic Review

**DOI:** 10.1371/journal.pone.0028631

**Published:** 2011-12-16

**Authors:** Marjan Askari, Peter C. Wierenga, Saied Eslami, Stephanie Medlock, Sophia E. de Rooij, Ameen Abu-Hanna

**Affiliations:** 1 Department of Medical Informatics, Academic Medical Center, Amsterdam, The Netherlands; 2 Department of Clinical Pharmacy, Academic Medical Center, Amsterdam, The Netherlands; 3 Department of Internal and Geriatric Medicine, Academic Medical Center, Amsterdam, The Netherlands; Marienhospital Herne - University of Bochum, Germany

## Abstract

**Background:**

Care of the elderly is recognized as an increasingly important segment of health care. The Assessing Care Of Vulnerable Elderly (ACOVE) quality indicators (QIs) were developed to assess and improve the care of elderly patients.

**Objectives:**

The purpose of this review is to summarize studies that assess the quality of care using QIs from or based on ACOVE, in order to evaluate the state of quality of care for the reported conditions.

**Methods:**

We systematically searched MEDLINE, EMBASE and CINAHL for English-language studies indexed by February 2010. Articles were included if they used any ACOVE QIs, or adaptations thereof, for assessing the quality of care. Included studies were analyzed and relevant information was extracted. We summarized the results of these studies, and when possible generated an overall conclusion about the quality of care as measured by ACOVE for each condition, in various settings, and for each QI.

**Results:**

Seventeen studies were included with 278 QIs (original, adapted or newly developed). The quality scores showed large variation between and within conditions. Only a few conditions showed a stable pass rate range over multiple studies. Overall, pass rates for dementia (interquartile range (IQR): 11%–35%), depression (IQR: 27%–41%), osteoporosis (IQR: 34%–43%) and osteoarthritis (IQR: 29–41%) were notably low. Medication management and use (range: 81%–90%), hearing loss (77%–79%) and continuity of care (76%–80%) scored higher than other conditions. Out of the 278 QIs, 141 (50%) had mean pass rates below 50% and 121 QIs (44%) had pass rates above 50%. Twenty-three percent of the QIs scored above 75%, and 16% scored below 25%.

**Conclusions:**

Quality of care per condition varies markedly across studies. Although there has been much effort in improving the care for elderly patients in the last years, the reported quality of care according to the ACOVE indicators is still relatively low.

## Introduction

The elderly population forms a precarious group characterized by multimorbidity, frailty and polypharmacy, leading to more complex care [1], [2]. Studies have shown that elderly patients do not receive the care that is known to be appropriate for them [3], [4]. It is postulated that there is much room for improvement of the quality of care for this group [5].

Efforts have been made to explore where, when and for which conditions quality deficiencies exist in order to know where improvements are needed. Measurement sets like HEDIS, with 75 measures across eight domains of care, have been developed to assist in assessing the quality of care. In addition criteria such as the Beers criteria were suggested to map the use of inappropriate medication for the elderly [6], [7]. The Assessing Care of Vulnerable Elders (ACOVE) quality indicator (QI) set was developed in the year 2000 by Rand Healthcare and the UCLA [8], [9] as a comprehensive method for assessing the quality of care of vulnerable elderly patients. Iterative expert panel meetings with review of the relevant evidence were used to generate a set of indicators to assess the quality of the process of care, rather than outcomes. RAND researchers postulate that these QIs represent minimal care rather than optimal care for the vulnerable elderly population, and are meant to assess and ultimately improve the quality of care [8], [9]. The resulting set consists of explicitly phrased IF-THEN clinical rules with comprehensive coverage of general medical and geriatric conditions, including comorbidities. These rules are intended to evaluate, by means of gauging adherence to the rules, the extent to which the care being delivered meets minimal standards of quality. The following is an example of an ACOVE indicator (or rule): “IF a vulnerable elder reports a history of two or more falls (or one fall with injury) in the previous year, THEN there should be documentation of a basic fall history (circumstances, medications, chronic conditions, mobility, alcohol intake) within three months of the report (or within four weeks of the report if the most recent fall occurred in the previous four weeks”). ACOVE-1 represents the first original set of QIs. The second phase of ACOVE (ACOVE-2) aimed at evaluating various interventions in primary care practices in order to improve care, but the QI set was not changed. The ACOVE-3 QI set is an updated and expanded set of QIs including five new conditions: COPD, colorectal cancer, breast cancer, sleep disorders, and benign prostatic hypertrophy.

Because ACOVE QIs or adaptations thereof have been used for over a decade for the assessment of quality of care, the opportunity now exists to synthesize the available evidence for the quality of care of a multitude of conditions in various settings. This paper reviews the studies that assessed the quality of care for elderly patients using ACOVE (-based) QIs in order to evaluate the state of the quality of care for the reported conditions.

## Methods

### Data sources and searches

Studies were identified by searching MEDLINE (via Scopus and PubMed), CINAHL and EMBASE by using the following search query:

ACOVE OR (“assessing care” AND (vulnerable OR frail*))

### Study Selection and Data Extraction

Relevant articles were included which used ACOVE QIs or adaptations of ACOVE QIs to assess the quality of care, and were published in the English language after the introduction of the ACOVE-1 set in 2001. Opinion papers, editorials, letters and congress abstracts were excluded. The last search was performed at the beginning of February 2010. Two reviewers (MA, PW) independently examined the collected studies in two rounds. The first round consisted of critically reading the title, abstract, and keywords. Studies selected in the first round had the full text reviewed in the second round. In the second round, we carefully checked the objectives of the studies and included those papers that used the ACOVE QIs (set 1, 2 or 3) or adaptations of those QIs to assess the quality of care of elderly patients. One investigator screened citations to identify additional candidate articles. In each round, disagreements between the two reviewers were resolved by consensus. If the two reviewers were unable to reach consensus a third reviewer was involved (AA) to make a final decision. Inter-rater agreement was measured by Cohen's kappa.

Using a structured extraction form, the two reviewers independently extracted the following information from the included studies: study characteristics (e.g., author, type of study, year), objectives, results, conclusion, QIs used, and conditions assessed by the QIs (see appendix S1 and checklist S1).

### Data Analysis and Synthesis

The results and conclusions of the included studies were evaluated to gain an overall picture of the quality of care for the elderly as measured by the ACOVE QIs. When possible, the results of the studies were combined, e.g. by extracting QI pass rates in each setting for each condition.

We analyzed the data at three levels: (1) conditions across studies, (2) conditions within distinct settings in studies, and (3) QIs across studies. At level (1), we extracted for each condition the reported QI-pass rates from each study. We then reported the low- and high-scoring conditions irrespective of setting. Interquartile ranges were provided where it was possible to do so (when more than four numbers were available).

To identify the proportion of high-scoring QIs per *condition* we also calculated the number and proportion of unique QIs with a mean score above 50% for each condition among all studies addressing that condition. For similar QIs among studies, their pass rates were first averaged. For example, consider two studies, one applying four QIs and the other three QIs for the same specific condition, of which two QIs are identical. We first average the pass rates for each of the two common QIs and obtain in total five pass rates for the unique QIs for the given condition in the two studies. Suppose that the (mean) scores of these QIs were 30%, 35%, 40%, 55%, and 60%, then we have a proportion of two out of five QIs with a mean score above 50%. We considered two QIs as similar if they appeared to have an identical or comparable content or intent. Our matching criteria allowed for differences in targeted patient population, time frame, level of specification and small textual differences in the QI contents. The most important differences in the phrasing of QIs are highlighted in the available supplemental table S1. In the case of interventional studies, we used the QI scores of the control group for this analysis.

At level (2) the dimension of a setting was added to the analysis, to increase homogeneity between them. Specifically, we considered conditions in the same setting among the various studies. We identified per setting all conditions that had a mean score <35% or >65% in any study. This helps focus attention to the low and high scoring conditions per setting.

At level (3) we synthesized evidence for QIs regardless of study or setting. For each QI we obtained its mean score (i.e. mean pass rate) across studies. Then we calculated the percentage of QIs having mean score below or above 50% (and below 25% and above 75%).

The list of all QIs used in the included studies was compared to the complete list of the original ACOVE-1 QIs in order to identify QIs that were not assessed in any included study.

## Results

**Figure 1:**
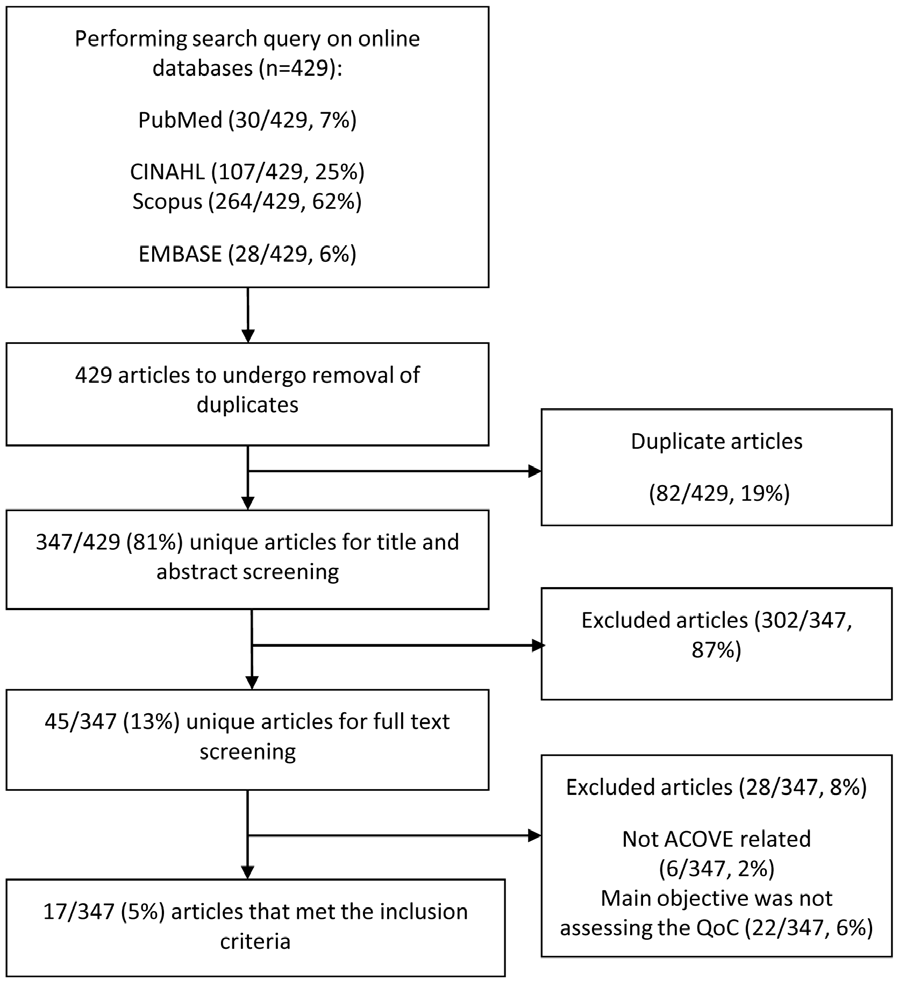
Article selection flow diagram – QoC: Quality of Care.

The database search resulted in 347 articles. Screening the titles and abstracts yielded 45 candidate articles for inclusion, of which 17 were included after full-text review [10]–[26]. Figure 1 (Diagram S1) shows the article selection flow diagram. Inter-rater agreement was high with Kappa of 0.76, where only five papers (5/347, 1%) necessitated the involvement of the third reviewer. Screening of the bibliographies yielded no additional studies for inclusion.

### Study characteristics

Nine of the seventeen studies (53%) assessed the overall quality of care or focused on a specific domain of care. Eight studies assessed care for a specific condition. Table 1 shows the study domains (“Overall quality of care”, “Specific domain of care”, and “Specific condition”).

**Table 1:** Study domains (“Overall quality of care”, “Specific domain of care”, and “Specific condition”).

		Domain name	
References	Overall quality of care	Specific domain of care	Specific condition
Zingmond DS et al.[^15^]	X		
Steel N, et al.[^17^]	X		
Wenger NS, et al.[^21^]	X		
Zingmond DS, et al. [^25^]	X		
Wenger NS, et al.[^26^]		Geriatric care	
Arora VM, et al.[^24^]		Quality of hospital care	
Mikuls TR, et al.[^19^]		Pharmacologic care	
Higashi T, et al.[^23^]		Pharmacologic care	
Spinewine A, et al.[^14^]		Appropriateness of prescribing or underuse	
Cadogan MP, et al.[^11^]			Management and detection of pain
Chodosh J, et al.[^22^]			Management and detection of pain
Rubenstein LZ, et al.[^12^]			Falls and instability
Asch SM, et al.[^13^]			Congestive heart failure care
Ganz DA, et al.[^16^]			Osteoarthritis
Bates-Jensen BM, et al.[^18^]			Pressure ulcer care
Schnelle JF, et al.[^10^]			Urinary incontinence
Gnanadesigan N, et al.[^20^]			Urinary incontinence

Eight studies used the original ACOVE QIs [12], [14], [16], [20]–[23], [26], all referring to the ACOVE-1 set, of which one [26] was performed during the second phase of ACOVE. The remaining nine studies used an adaptation of ACOVE QIs or newly developed ACOVE-like QIs [10], [11], [13], [15], [17]–[19], [24], [25].

Fifteen out of seventeen studies (88%) were done in the US and two studies were done in Europe (one in Belgium and one in the UK). Four studies [12], [20], [22], [23] used the same data sample as the study by Wenger et al. [21]. Three of them [20], [22], [23] used different QIs or a different number of QIs than those used by Wenger et al. The differences are marked in the supplemental table S1. The QIs in these four studies that overlapped with the Wenger et al. study sometimes had small differences in the pass rates, as compared to those reported in Wenger et al., perhaps related to how eligibility for QIs was determined. We treat these studies as dependent, meaning that the QIs in those studies are counted only once in the analysis. The population in the included studies ranged from age 50 and older, to age 75 and older. All but three studies [13], [17], [19] included only patients aged 65 and older. Vulnerable elderly patients were the explicit target population in only six studies, five of which used the same patient sample, as described in the previous paragraph.

### Quality of care

Table S2 shows the pass rates and number of QIs for all of the specific conditions. The number of QIs used per study ranged between three and 207, and between one to 43 QIs per condition or domain of care (e.g. pharmacological care). The quality of care for each condition varied greatly in the included studies. Furthermore, when the quality of care for a single condition was assessed in multiple studies, this was also highly variable. Only few conditions showed a stable range of pass rates over multiple studies. Overall, the quality scores for dementia (interquartile range: 11%–35%), depression (interquartile range: 27%–41%), osteoporosis (interquartile range: 34%–43%) and osteoarthritis (interquartile range: 29–41%) were notably low, regardless of setting. Medication management and use (range: 81%–90.30%), hearing loss (range:77%–78.9%) and continuity of care (range:76%–80%) on the other hand, scored relatively higher than other conditions.

From the seventeen studies, four studies focused on nursing home residents [10], [11], [18], [25], five on managed care plans [12], [20]–[23], two on patients admitted to hospital [14], [24] and four on primary care patients [15], [17], [19], [26]. Two studies had mixed settings [13], [16]. Because only one or two studies within a setting assessed the same condition, calculating a mean per setting for each condition was not meaningful, hence we report the score of the corresponding studies. Table 2 shows the high and low scoring conditions within each setting.

**Table 2:** High and low scoring conditions within each setting.

Settings	Low scoring conditions	High scoring conditions
**Nursing home**	Dementia (9%)	Medication management (90%)
	Depression (16%)	End-of life care (89%)
	Stroke (20%)	Malnutrition (77%)
	Ischemic heart disease (22%)	
	Heart failure (23)	
	Osteoarthritis (26 and 26%–46%)	
	Osteoporosis (27%)	
**Managed care settings**	End-of-life care (9%)	Stroke (82%)
	Osteoarthritis (31%)	Medication use (81%)
	Depression (31%)	Continuity of care (80%)
	Falls (34%^*^)	Vision (79%)
		Hypertension (77%)
		Hearing loss (77%)
		Heart failure (71%)
		Screening & prevention (67%)
**Primary care**	Osteoarthritis (29%)	Medication management (83%)
		Pain management (78%)
		Hearing loss (79%)
		Continuity of care (76%)
		Smoking (74%)
		Diabetes (74%)
		Hypertension (72%)
		Stroke (65%)
**Hospital**	Dementia (31%)	Falls (83%)
		Hospital care (82%)

In the hospital setting, the quality of care for falls was higher than in other settings. Diabetes scores were average in all settings; nevertheless the score in the UK primary care setting was higher than in other health care settings. In the only study in the primary care setting of the UK, the pass rates for ischemic heart disease, diabetes, depression, hypertension, osteoporosis, urinary incontinence, stroke and vision care were all higher than in the US. There were three studies in primary care in the US, which used a range of three to 43 QIs to measure quality of care in one to 22 conditions, while the single UK study used 32 QIs for 12 conditions. Therefore, in terms of numbers of QIs, more QIs were used in the US studies than in the UK study.

### Results per QI

Table 3 shows the QIs that were most frequently used (more than four times), regardless of setting. For the sake of brevity, Table 3 uses an abbreviated version of the QIs. The supplemental table S1 shows the full text of QIs used in all studies.

**Table 3:** Most frequently used QIs.

Quality indicators	Number of unique times that QI was used
IF analgesia required THEN NOT meperidine	4[11], [15], [21], [25]
IF heart failure and LV ejection fraction ≤40% THEN ACE inhibitor or receptor blocker	4[13], [14], [21], [25]
IF newly diagnosed dementia THEN measure vitamin B12 and thyroid-stimulating hormone	4[15], [21], [25], [26]
IF depression, THEN antidepressant treatment, psychotherapy, or electroconvulsive therapy within 2 weeks	4[15], [17], [21], [25]
IF diabetes THEN yearly HbA1C	4[15], [17], [21], [25]
IF new heart failure THEN evaluation of LV ejection fraction	4[13], [15], [21], [25]
IF established CHD and LDL cholesterol level >130 mg/dL THEN cholesterol-lowering medication	4[13], [15], [21], [25]
IF female has a new diagnosis of osteoporosis, THEN hormone replacement therapy, bisphosphonates, a selective estrogen receptor modulator or calcitonin within 3 months	4[15], [17], [21], [25]

VE: Vulnerable elderly; CHD: Chronic heart disease; LDL: Low density lipoprotein; LV: Left ventricular.

When comparing the QIs that were used with the entire original ACOVE-1 QI set, we found that 35 ACOVE-1 QIs were not used in any of the studies. All QIs for 10 conditions (diabetes, falls, hypertension, ischemic heart disease, osteoarthritis, osteoporosis, pain management, pressure ulcers, preventive care and urinary incontinence) were used in at least one study. The 35 unused QIs were distributed among the 14 remaining conditions, with a range of 7% (one out of 14 heart failure QIs) to 44% (four out of nine QIs for hospital care), and a median of 28% unused QIs within a condition. The list of the 35 QIs is provided in the supplemental table S1.

From the 278 QIs that were used in the included studies (original, adapted or newly developed for the new conditions) 16% (46 QIs) scored below 25%, 50% (141 QIs) had mean pass rates below 50%, 44% (121 QIs) above 50% and 22% (62 QIs) above 75%. Sixteen QIs were reported in the included studies as having no eligible patients, therefore the pass rates could not be calculated.

Table S2 reports on the number of QIs used in the studies that had pass rates above 50%. Seventy-five percent of the QIs pertaining to medication management, hearing loss and continuity of care scored above 50%, making them the highest-scoring conditions.

## Discussion

In this systematic review we described the results of 17 research papers using the ACOVE quality indicators to assess the quality of care. The assessment of care was performed in a variety of care settings, in several different elderly patient populations and for multiple conditions. Due to this heterogeneity and the fact that the studies used different subsets of the ACOVE QIs or adaptations thereof, the results of the studies cannot be directly compared and hence a quantitative meta-analysis is not justified. However, considering that many studies assessed the quality of care for multiple conditions simultaneously and 50% of the QIs had a pass rate below 50%; some general conclusions can be drawn about areas to which improvement initiatives should be focused. An overall conclusion is that there is much room for care improvement for the elderly population.

Individual studies have already shown the need for greater focus on elderly care [5], [27], [28]. This finding is supported by our review. Based on the included studies the overall quality scores for dementia, depression, osteoporosis and osteoarthritis were notably low. In addition to the conditions above, hypertension, ischemic heart disease, pressure ulcer, pain management, falls and urinary incontinence scored below 50% at the QI level.

In the interest of maintaining a good quality of life for elderly patients it is very important to treat geriatric conditions, and it may even be unethical to ignore this need. Although care for many conditions showed deficiencies, geriatric conditions like dementia and falls seem to show greater deficiencies than others. This may be due to less attention to and awareness of the need for good treatment of age-related and geriatric conditions, or poor identification of these conditions [29]–[32]. The deficiencies may also be caused by insufficient teaching of the skills and expertise needed to perform these processes of care [33]. This review cannot conclude which factors are more influential, and future studies are needed to uncover the reasons why some QIs have low pass rates.

On the other hand, medication management and use, hearing loss and continuity of care, scored markedly higher than other conditions regardless of the setting and patient population and regardless of which QIs were used to assess them. This could be due to the increased attention to medication management in general, or partly attributable to chance due to the relatively low number of studies including these conditions. Although based on only one study, quality of care for falls in the hospital setting scored markedly higher than in other settings. This difference may be explained by fewer QIs being used in the hospital study and differences in the QIs that were used in the individual studies, or by increased attention to falls in hospitals and the more intensive care given to hospitalized patients compared to other settings [34], [35]. There was only one UK study in the primary care setting compared to three US studies. Although different QIs were used, the care for ischemic heart disease, diabetes, depression, hypertension, osteoporosis, urinary incontinence, stroke and vision care had better quality in the UK primary care setting compared to the US. It is plausible that this is due to differences in diagnoses and treatment of these conditions between the countries, or a different prevention program [36], [37]. This finding does not warrant general conclusions about the differences in quality of care between the countries, and more studies are needed.

Although comparison of scores per setting was based on limited studies and QIs, it may reveal the need for extra attention to the conditions that form good candidates for quality improvement. These are the conditions that had mean scores below 50%. In managed care settings these conditions are: osteoarthritis, depression, urinary incontinence (UI), falls, dementia, end-of life care, malnutrition, pressure ulcer care, and pneumonia care. In nursing homes, dementia, depression, diabetes, falls, stroke, ischemic heart disease, heart failure, osteoarthritis, osteoporosis, atrial fibrillation, vision and hypertension had consistently low scores. Finally, in primary care, dementia, UI, falls, osteoarthritis and vision care show room for improvement.

According to the ACOVE indicators and the studies identified by our review, it appears that the quality of care for the elderly is low. However, we can only draw limited conclusions from these studies, for several reasons. First, although the QIs are generally evidence–based and have been developed in multiple Delphi rounds using expert panels, it is still possible that individual physicians will debate the content of specific QIs. Although the QIs are conjectured to represent minimal care, it is possible that low pass rates may represent legitimate differences of medical opinion. Second, undocumented patient refusal of the offered care could lead to a lower measured pass rate. Various studies, however, have taken this aspect into account and counted an indicator as passed when a patient refused the indicated care or when a contraindication existed. Third, identifying the vulnerable elderly (VE) is difficult and, probably due to this difficulty, the majority of the studies did not distinguish between the vulnerable elderly and the general elderly population. Since ACOVE was designed for a vulnerable elderly population, this can lead to a biased score. Fourth, the reason for selecting a certain number and type of QIs for the assessment of care for a specific condition was not always clearly described in the studies. Difficulty in the assessment of some of the QIs could have lead to omitting these QIs from the assessment of that condition and consequently to selection bias. This can result in an incomplete picture of the quality of care of patients for the specific condition. Poor record-keeping can influence, positively or negatively, the pass rates of various QIs. It is plausible that correct care was performed but not documented, which can lead to lower pass rates. On the other hand, poor-record keeping for the “IF” part of a rule renders the rule as inapplicable and hence failure to provide the correct care will go undetected. Irrespective of the ability to measure QI pass rates, lack of documentation can be an indicator of poor quality because it hampers continuity of care and contributes to miscommunication [23]. Fifth, variation in scores of quality of care could be caused by either variation in the number of QIs used per study or by the fact that QIs focused on different aspects of care for a specific condition. Moreover, variation in the study sample sizes can cause differences in the pass rates per condition. A smaller study population gives more opportunity for chance findings. We suggest that future studies should explicitly mention and discuss these factors.

To our knowledge, this is the first review on assessing quality of care of elderly patients using the ACOVE criteria. Although our literature search has been systematic and extensive in order to give a complete overview of the studies using ACOVE for assessing the elderly population care, it is still plausible that some articles were missed.

### Conclusion and recommendation

Our results showed that despite the large efforts that have been expended in improving the care for elders in the last years, quality of care for elderly patients as measured by the ACOVE criteria is still poor. This is particularly worrisome as the ACOVE criteria are meant to represent a minimal standard of care for the vulnerable elderly population, although not all of the included studies included a measure of vulnerability in their inclusion criteria. The majority of the assessed conditions and domains of care seem to merit further quality improvement effort and/or a better understanding of why some QIs have low pass rates.

The ACOVE QI set provides a promising and uniquely comprehensive method for assessing the quality of care of elderly patients. However, to improve the extent to which studies can be compared, two important factors should be taken into consideration. First, researchers should strive to assess all QIs for a domain of interest, instead of a small selection thereof. This is especially important because there may be an association between ease of measuring a QI and its score. Second, should one require the adaptation of original QIs, then one should measure the same underlying concept implied by the original QIs and explicitly report on the nature of the adaptation.

## Supporting Information

Table S1 QI: quality indicator; *: No patients were eligible for the premise of the QI. Co: condition. Note: we rounded the percentages. !: QIs that were scored in a study, using the Wenger data set. †: QIs that had different pass rates than in Wenger et al. study. ‡: Those QIs that were scored in the studies using the same data set as Wenger et al, but were not scored in Wenger. -: The QIs that were not used in any study.
(DOC)

Table S2 **Measured mean pass rate of QIs per condition and proportion of unique (matched) QIs with mean score above 50% per condition.** ‡: The pass rate is reported for both delirium and dementia. †: These QIs were about physical functioning. QI: quality indicator, VE: vulnerable elder(s), NH: Nursing home, PC: Primary care, PIM: Prescribing indicated Mediations, AIM: Avoiding Inappropriate Medication, ECD: Education, Continuity, and Documentation, MM: Medication Monitoring, GEM: Geriatric Evaluation and Management, CHF: Chronic Heart Failure, IHI BTS: Institute of Healthcare Improvement's Breakthrough Series, PC: Primary Care. *: The same patient population and dataset was used as in the Wenger et al. study [21], for these common QIs we only considered the pass rates reported in [21] for our analysis.
(DOC)

Appendix S1 **Table of extracted data.** ACOVE: Assessing Care Of Vulnerable Elders; VE: Vulnerable Elder; NH: Nursing Home; QoC: Quality Of Care; QI: Quality Indicators; IHI BTS: Institute of Healthcare Improvement's Breakthrough Series; CHF: Chronic Heart Failure; GEM: Geriatric Evaluation and Management; MAI: Medication Appropriateness Index; DM; Diabetes Mellitus; PU: Pressure Ulcer; MDS: Minimum Data Set; CI: Cognitive Impairment; UI: Urinary Incontinence; PIM: Prescribing Indicated Medication; AIM: Avoiding Inappropriate Medication; ECD: Education, Continuity, and Documentation; MM: Medication Monitoring; QoL: Quality of Life; RA: Rheumatoid Arthritis; AF: Atrial Fibrillation; OA: Osteoarthritis; 1:# = Number of quality indicators used.
(DOC)

Checklist S1 **PRISMA checklist.**
(DOC)

Diagram S1 **PRISMA Flow Diagram.**
(DOC)
