# Supplementary material for: Assessing Quality of Care of Elderly Patients Using the ACOVE Quality Indicator Set: A Systematic Review
Source: PLoS One. 2011 Dec 16;6(12):e28631. doi: 10.1371/journal.pone.0028631 (PMC3241679; doi:10.1371/journal.pone.0028631)
Supplement: Table S1 — QI: quality indicator; *: No patients were eligible for the premise of the QI. Co: condition. Note: we rounded the percentages. !: QIs that were scored in a study, using the Wenger data set. †: QIs that had different pass rates than in Wenger et al. study. ‡: Those QIs that were scored in the studies using the same data set as Wenger et al, but were not scored in Wenger. -: The QIs that were not used in any study. (DOC) [file pone.0028631.s001.doc]

Supplemental table S1

QI: quality indicator; *: No patients were eligible for the premise of the QI. Co: condition. Note: we rounded the percentages.

!: QIs that were scored in a study, using the Wenger data set .†: QIs that had different pass rates than in Wenger et al. study. ‡: Those QIs that were scored in the studies using the same data set as Wenger et al, but were not scored in Wenger. -: The QIs that were not used in any study.

| **Condition** | **Quality indicators** | **Number of studies using the QI** | **Mean[Median(Min, Max)]** |
| --- | --- | --- | --- |
| **Falls and mobility** | All vulnerable elders should have documentation that they were asked at least annually about the occurrence of recent falls^!^ | 1 | 25 |
|  | All vulnerable elders should have documentation that they were asked about or examined for the presence of balance and gait disturbances at least once^! †^ | 1 | 49[48.5(48;49)] |
|  | IF a (vulnerable elder /person aged 65 or older -Steel-) reported two or more falls in the past year or a single fall with injury requiring treatment, Then (there should be documentation that a basic fall history was performed / the physician should take a basic fall history -Steel-)^!^ | 3 | 44[49(34;49)] |
|  | IF a vulnerable elder reported two or more falls in the past year, or a single fall with injury requiring treatment, Then there should be documentation that a basic fall physical examination was performed^!^ | 2 | 8[8(3;12)] |
|  | IF a vulnerable elder reported two or more falls in the past year or a single fall with injury requiring treatment, Then there should be documentation of specific diagnostic and therapeutic recommendations^!^ | 1 | 30 |
|  | IF a person aged 65 or older reported 2 or more falls in the past year, or a single fall with injury requiring treatment, THEN the patient should be offered a multidisciplinary falls assessment. | 1 | 38 |
|  | IF a vulnerable elder reports or is found to have new or worsening difficulty with ambulation, balance, or mobility, THEN a basic gait, mobility, and balance evaluation should be performed within 6 months that results in specific diagnostic and therapeutic recommendations specific diagnostic and therapeutic recommendations^!^ | 2 | 22[22(20;33)] |
|  | IF a vulnerable elder demonstrates decreased balance or proprioception or increased postural sway, THEN an evaluation for an assistive device performed (Wenger 2009) and an appropriate exercise program should be offered^! †^ | 2 | 37[37(12;62)] |
|  | IF a vulnerable elder is found to have problems with gait, strength, or endurance, THEN an exercise program should be offered^!^ | 3 | 58[70(30;71)] |
|  | Home safety evaluation | 1 | 4 |
|  | treat balance problem | 1 | 21 |
|  | Treat strength or gait problem | 1 | 58 |
|  | NH resident with new balance difficulty should receive PT or an assistive device | 1 | 34 |
| **Dementia** | IF a vulnerable elder is admitted to a hospital or is new to a physician practice, THEN multidimensional assessment of cognitive ability and assessment of functional status should be documented. | 2 | 53[53(52;53)] |
|  | IF a vulnerable elder is admitted to a hospital or is new to a physician practice, THEN there should be an assessment of functional status. | 1 | 18 |
|  | IF a (vulnerable elder / NH resident -Zingmond09-) has newly diagnosed dementia, THEN serum levels of vitamin B12 and thyroid-stimulating hormone should be measured. | 4 | 14[14(2;25)] |
|  | IF a vulnerable elder has (mild to moderate Alzheimer disease / newly diagnosed dementia - Zingmond07-), THEN the treating physician should (discuss treatment / treat -Zingmond07-) with a cholinesterase inhibitor with the patient and the primary caregiver (if available). | 3 | 44[44(13;75)] |
|  | IF a vulnerable elder with dementia has a caregiver (and, if capable, the patient assents), THEN the physician should discuss or refer the patient and caregiver for discussion about patient safety, provide education on how to deal with conflicts at home, and inform them about community resources for dementia | 2 | 24[24(21;26)] |
|  | IF a vulnerable elder has dementia, THEN he or she should be screened for depression during the initial evaluation. | 3 | 47[50(31,2;60)] |
|  | IF a vulnerable elder has newly diagnosed dementia, THEN the diagnosing physician should advise the patient not to drive a motor vehicle or request that the Department of Motor Vehicles (or an equivalent agency) retest the patient's ability to drive, or refer to a drivers safety course that includes assessment of driving ability | 2 | 25[25(0;50)] |
|  | IF a vulnerable elder with dementia has cerebrovascular disease, THEN he or she should be offered appropriate stroke prophylaxis | 1 | 100 |
|  | IF a (vulnerable elder / NH resident -Zingmond09-) with dementia has depression, THEN he or she should be treated for the depression (with pharmacologic therapy or mental health referral -Zingmond07-) | 3 | 20 |
|  | If a vulnerable elder with dementia is to be physically restrained in the hospital, then the target or safety issue justifying use of restraints must be identified to the consenting person and documented in the chart | 1 | 100 |
|  | If a vulnerable elder is physically restrained and the target behavioural disturbance requiring restraint is identified, then the healthcare team should include methods other than physical restraints in the care plan | 1 | 69 |
|  | Classify type of dementia in medical record | 1 | 40 |
|  | Objective mental status test in new dementia | 1 | 60 |
|  | Evaluation need for help with functional activities | 1 | 52 |
|  | Evaluate decision-making capacity | 1 | 27 |
|  | IF a vulnerable elder presents with symptoms of dementia, THEN the physician should review the patient’s medication list for initiation of medications that might correspond chronologically to the onset of dementia symptoms. | 0 | - |
|  | IF a vulnerable elder presents with symptoms of dementia that correspond in time with the initiation of new medications, THEN the physician should discontinue or justify the necessity of continuing these medications. | 0 | - |
|  | IF a vulnerable elder has signs of dementia and focal neurologic findings that suggest an intracranial process, THEN he or she should be offered neuroimaging (brain computed tomography or magnetic resonance imaging). THEN he or she should be offered neuroimaging (brain computed tomography or magnetic resonance imaging). | 0 | - |
|  | IF a vulnerable elder is placed in physical restraints, THEN each of the following measures should be enacted: 1) Consistent release from the restraints at least every 2 hours; 2) Face-to-face reassessment by a physician or 3) Observation at least every 15 minutes, and more frequently if indicated by the patient’s condition, while the nurse at least every 4 hours and before renewal of the restraint order; patient is in restraints; 4) Interventions every 2 hours (or as indicated by patient’s condition or needs) related to nutrition, hydration, personal hygiene, toileting, and range of motion exercises. | 0 | - |
| **Continuity of Care** | ALL vulnerable elders should be able to identify a physician or a clinic that they would call when in need of medical care or should know the phone number or other mechanism by which they can reach this source of care | 1 | 100 |
|  | IF an outpatient, vulnerable elder is started on a new prescription medication, and he or she has a follow-up visit with the prescribing physician, THEN the medical record at the follow-up visit should document 1 of the following: 1) the medication is being taken, 2) the physician asked about the medication, or 3) the medication was not started because it was not needed or because it was changed.^!^ | 1 | 66 |
|  | IF a vulnerable elder is under the outpatient care of more than 2 physicians, and 1 physician prescribed a new prescription medication or a change in medications, THEN subsequent medical record entries by the non-prescription physician should acknowledge the medication change.^!^ | 1 | 42 |
|  | IF a vulnerable elder is discharged from a hospital to home, and he or she received a new prescription medication or a change in medication before discharge, THEN the outpatient medical record should document or acknowledge the medication change within 6 weeks of discharge.^!^ | 1 | 55 |
|  | IF a vulnerable elder is discharged from a hospital to home or to a nursing home, and the transfer form or discharge summary indicates that a test result is pending, THEN the outpatient or nursing home medical record should include the test result within a 6 weeks of hospital discharge. | 1 | 71 |
|  | IF a vulnerable elder is discharged from a hospital to home or to a nursing home, and the hospital medical record specifies a follow-up appointment for a physician visit or a treatment (e.g., physical therapy or radiation oncology), THEN the medical record should document that the visit or treatment took place or that it was postponed or not needed | 1 | 90 |
|  | IF a vulnerable elder is discharged from a hospital to home or to a nursing home, THEN there should be a discharge summary in the outpatient physician or nursing home medical record within 6 weeks. | 2 | 59[59(41,76)] |
|  | IF a vulnerable elder is deaf or does not speak English, THEN an interpreter or translated materials should be employed to facilitate communication between the vulnerable elder and the health care provider | 1 | [*] |
|  | IF an outpatient vulnerable elder is referred to a consultant physician, THEN the reason for consultation should be documented in the consultant’s note. | 0 | - |
|  | IF an outpatient vulnerable elder is referred to a consultant and subsequently visits the referring physician after the visit with the consultant, THEN the referring physician’s follow-up note should document the consultant’s recommendations, or the medical record should include the consultant’s note, within 6 weeks or at the time of the follow-up visit, whichever is later. | 0 | - |
|  | IF the outpatient medical record documents that a diagnostic test was ordered for a vulnerable elder, THEN the medical record at the follow-up visit should document one of the following: 1) the result of the test, 2) the test was not needed or reasoned why it will not be performed, or 3) the test is still pending. | 0 | - |
|  | IF a vulnerable elder is transferred between emergency departments or between acute care facilities, THEN the medical record at the receiving facility should include medical records from the transferring facility or should acknowledge transfer of such medical records. | 0 | - |
|  | IF a vulnerable elder is discharged from a hospital to home or to a nursing home, THEN there should be a discharge summary in the outpatient physician or nursing home medical record within 6 weeks. | 0 | - |
| **Depression** | IF a vulnerable elder presents with new onset of one of the following symptoms: sad mood, feeling down, insomnia or difficulties with sleep, apathy or loss of interest in pleasurable activities, complaints of memory loss, unexplained weight loss greater than 5% in the past month or 10% over 1 year, or unexplained fatigue or low energy, THEN the patient should be asked about or treated for depression, or referred to a mental health professional within 2 weeks of presentation. | 1 | 26 |
|  | IF a vulnerable elder presents with onset or discovery of one of the following conditions: stroke, myocardial infarction, dementia, malignancy (excluding skin cancer), chronic pain, alcohol or substance abuse or dependence, anxiety disorder, or personality disorder, THEN the patient should be asked about or treated for depression, or referred to a mental health professional within 2 months of diagnosis of the condition. | 1 | 0 |
|  | IF a (vulnerable elder / person aged 50 or older -Steel-) receives a diagnosis of a new depression episode, THEN 4 (the medical record should document / the diagnosing physician should ask -Steel-) on the day of diagnosis (the presence or absence of suicidal ideation / had any thoughts about suicide -Steel-) (and psychosis (consisting of, at a minimum, auditory hallucinations or delusions). -deleted Steel-) | 2 | 23[23(0;45)] |
|  | IF a vulnerable elder has thoughts of suicide, THEN the medical record should document, on the same date, that the patient either has no immediate plan for suicide or that the patient was referred for evaluation for psychiatric hospitalization. | 1 | [*] |
|  | IF a (vulnerable elder / NH resident -Zingmond09-/person aged 50 or older -Steel-) is diagnosed with (clinical -Steel-) depression, THEN (Treat with pharmacologic therapy or mental health referral within 2 wk -Zingmond07- / should be treated within 2 wks of diagnosis -Zingmond09- / antidepressant treatment, psychotherapy, or electroconvulsive therapy should be offered within 2 weeks after diagnosis unless there is documentation within that period that the patient has improved, or unless the patient has substance abuse or dependence, in which case treatment may wait until 8 weeks after the patient is in a drug- or alcohol-free state) | 4 | 46[46(12;79)] |
|  | IF a (vulnerable elder / NH resident -Zingmond09-) is started on an antidepressant medication, THEN the following medications should not be used as first- (or second- -deleted Zingmond09-) line therapy: tertiary amine tricyclics (amitriptyline, imipramine, doxepin, clomipramine, trimipramine); MAOIs (unless atypical depression is present); benzodiazepines; or stimulants (except methylphenidate).^!^ | 3 | 92[90(89;97)] |
|  | IF a (vulnerable elder / NH resident -Zingmond09-) with a history of (cardiac disease / coronary artery disease -Zingmond09, Zingmond07-) is started on a tricyclic antidepressant, THEN (a baseline electrocardiogram should be obtained before initiation of or within 3 months before treatment / should have a baseline ECG performed -Zingmond09-) (exclusion: Pacemaker -Zingmond07-). | 3 | 24[30(0;43)] |
|  | IF a (vulnerable elder / patient with depression -Zingmond07-) is taking a serotonin reuptake inhibitor, THEN (an MAOI should not be used for at least 2 weeks after termination of paroxetine, sertraline, fluvoxamine, and citalopram or for at least 5 weeks after termination of fluoxetine / MAOI started a specified time after stopping SSRI -Zingmond07-). | 3 | 90[90(80,100)] |
|  | IF a (vulnerable elder / patient with depression -Zingmond07-) is taking an MAOI, THEN (he or she should not receive medications that interact with MAOIs for at least 2 weeks after termination of the MAOI / SSRI started a specified time after stopping MAOI -Zingmond07- ) | 3 | 66[66(32;100)] |
|  | NH resident taking an SSRI should have an appropriate washout period before starting an MAOI, and vice versa. |  | [*] |
|  | IF a vulnerable elder is being treated for depression, THEN at each treatment visit suicide risk should be documented, if he or she had suicidal ideation during a previous visit. | 1 | [*] |
|  | IF a (vulnerable elder / person aged 50 or older -Steel-) has no meaningful symptom response after 6 weeks of treatment, THEN 1 of the following treatment options should be initiated by the 8th week of treatment: Medication dose should be optimized or the patient should be referred to a psychiatrist (if initial treatment was medication), or mediation should be initiated (if initial treatment was psychotherapy alone -Steel-) or referral to a psychiatrist should be offered (if initial treatment was psychotherapy alone -deleted Steel-).^!^ | 2 | 41[50(22;78)] |
|  | IF a vulnerable elder responds only partially after 12 weeks of treatment, THEN 1 of the following treatment options should be instituted by the 16th week of treatment: Switch to a different medication class or add a second medication to the first (if initial treatment included medication), add psychotherapy (if the initial treatment was medication), try medication (if initial treatment was psychotherapy without medication), consider electroconvulsive therapy, or refer to a psychiatrist. | 1 | 25 |
|  | IF a (vulnerable elder / NH resident -Zingmond09-) (has responded to antidepressant medication / with new depression that improves with treatment -Zingmond09-), THEN he or she should be continued (on the drug at the same dose / that antidepressant -Zingmond09-) for at least 6 months (and should make contact with a clinician at least once (office visit or phone) during that time period. -deleted Zingmond09-) | 2 | 69 |
|  | NH resident with newly diagnosed depression should have TSH checked | 1 | 11 |
|  | IF a vulnerable elder receives a diagnosis of a new depression episode, THEN the medical record should document on the day of diagnosis the presence or absence of suicidal ideation and psychosis (consisting of, at a minimum, auditory hallucinations or delusions). | 0 | - |
|  | IF a vulnerable elder has depression with psychotic features (for example, auditory hallucinations, delusions) or has melancholic or vegetative depression with pervasive anhedonia, unreactive mood, psychomotor disturbances, severe terminal insomnia, and weight and appetite loss, THEN he or she should not be treated with psychotherapy alone, unless he or she is unable or unwilling to take medication. | 0 | - |
|  | IF a vulnerable elder has depression with psychotic features, THEN he or she should be referred to a psychiatrist and should receive treatment with a combination of an antidepressant and an antipsychotic, or with electroconvulsive therapy. | 0 | - |
|  | IF a vulnerable elder is being treated for depression with antidepressants, THEN the antidepressants should be prescribed at appropriate starting doses, and they should have an appropriate titration schedule to a therapeutic dose, therapeutic blood level, or remission of symptoms by 12 weeks. | 0 | - |
| **Diabetes** | IF a (vulnerable elder / NH resident -Zingmond09- / person aged 50 or older -Steel-) has diabetes, THEN his or her glycosylated hemoglobin level (or fructosamine -Steel-) should be measured at least every 12 months. | 4 | 59[61(32; 83)] |
|  | IF a vulnerable elder has an elevated glycosylated hemoglobin level, THEN he or she should be offered a therapeutic intervention aimed at improving glycemic control within 3 months if the glycosylated hemoglobin level is 9.0% to 10.9%, and within 1 month if the glycosylated hemoglobin level is 11% or greater. | 1 | 61 |
|  | IF a diabetic (vulnerable elder / person aged 50 or older -Steel-) (does not have established renal disease / without renal insufficiency -Zingmond07-) and is not receiving an ACE inhibitor or ACE receptor blocker, THEN he or she should receive an annual test for proteinuria. | 3 | 46[36(19;83)] |
|  | IF a diabetic (vulnerable elder / NH resident -Zingmond09-) has proteinuria, THEN (he or she should be offered / prescribe -Zingmond07-) therapy with an ACE inhibitor or ACE receptor blocker (exclusions: ACE/ARB exclusions -Zingmond07-).^!^ | 3 | 72[98(20,98)] |
|  | IF a vulnerable elder has diabetes, THEN his or her blood pressure should be checked at each outpatient visit. | 1 | 59 |
|  | IF a diabetic vulnerable elder has a glycosylated hemoglobin level of 10% or greater, THEN he or she should be referred for diabetic education at least annually | 1 | [*] |
|  | IF a diabetic vulnerable elder has elevated blood pressure, THEN he or she should be offered a therapeutic intervention to lower blood pressure within 3 months if blood pressure is 150 to 160/90 to 100 mm Hg or within 1 month if blood pressure is greater than 160/100 mm Hg. | 1 | 79 |
|  | ALL diabetic vulnerable elders should be offered daily aspirin therapy^.!^ | 2 | 50[ 50(41;60)] |
|  | IF a diabetic (vulnerable elder / person aged 50 or older -Steel-) has a fasting total cholesterol level of (240 g/dL / 5 mmol/L -Steel-) or greater, THEN he or she should be offered an intervention to lower cholesterol. | 2 | 90[90(88;92)] |
|  | IF a diabetic (vulnerable elder / NH resident -Zingmond09-) is not blind, THEN he or she should receive an (annual dilated eye examination performed by an ophthalmologist, optometrist, or diabetes specialist / eye examination every 2 yr -Zingmond07- / annual dilated eye exam -Zingmond09-) | 3 | 46[48(39;51)] |
|  | IF a diabetic person aged 50 or older has one additional cardiac risk factor (i.e., smoker, hypertension, hypercholesterolemia, or renal insufficiency/microalbuminuria), THEN he/she should be offered an ACE inhibitor or receptor blocker. | 1 | 48 |
|  | ALL diabetic persons aged 50 or older should have an annual examination of his/her feet. | 1 | 84 |
| **End of life care** | ALL (vulnerable elders / NH resident -Zingmond09-) should have (an advance directive -Zingmond09- / in their outpatient charts 1) an advance directive indicating the patient’s surrogate decision maker, 2) documentation of a discussion about who would be a surrogate decision maker or a discussion about a search for a surrogate, or 3) indication that there is no identified surrogate) | 2 | 44[44(4;84)] |
|  | IF a vulnerable elder with dementia, coma, or altered mental status is admitted to the hospital, THEN within 48 hours of admission the medical record should 1) contain an advance directive indicating the patient’s surrogate decision maker, 2) document a discussion about who would be a surrogate decision maker or a discussion about a search for a surrogate, or 3) indicate that there is no identified surrogate. | 1 | 25 |
|  | IF a vulnerable elder has a diagnosis of severe dementia, is admitted to the hospital, and survives 48 hours, THEN within 48 hours of admission, the medical record should document consideration of the patient’s previous preferences for care or that these could not be elicited or are unknown. | 1 | 100 |
|  | IF a vulnerable elder is admitted directly to the intensive care unit (from the outpatient setting or emergency department) and survives 48 hours, THEN within 48 hours of admission, the medical record should document consideration of the patient’s preferences for care or that these could not be elicited or are unknown | 1 | 17 |
|  | IF a vulnerable elder indicates during an interview that he or she would rather die than live permanently comatose, ventilated, or tube fed, THEN 1) the chart should document a discussion of life-sustaining treatment preferences, 2) the chart should contain an advance directive, or 3) the patient should indicate that he or she discussed this topic with the physician or does not wish to discuss this | 1 | 12 |
|  | IF a vulnerable elder has an advance directive in the outpatient, inpatient, or nursing home medical record or the patient reports the existence of an advance directive in an interview, and the patient receives care in a second venue, THEN 1) the advance directive should be present in the medical record at the second venue or 2) documentation should acknowledge its existence, its contents, and the reason that it is not in the medical record. | 1 | 25 |
|  | IF a vulnerable elder requires mechanical ventilation during a hospitalization (except short-term and postoperative mechanical ventilation), THEN the medical record should document within 48 hours of the initiation of mechanical ventilation the goals of care and the patient’s preference for mechanical ventilation or why this information is unavailable. | 1 | 100 |
|  | IF a vulnerable elder with decision-making capacity has orders written in the hospital or the nursing home to withhold or withdraw a particular treatment (e.g., a do-not-resuscitate order or an order not to initiate dialysis), THEN the medical record should document 1) patient participation in the decision or 2) why the patient chose not to participate in the decision. | 1 | 70 |
|  | NH resident should have aggressiveness of care treatment preferences followed | 1 | 96 |
|  | IF a noncomatose vulnerable elder is not expected to survive and a mechanical ventilator is withdrawn or intubation is withheld, THEN the patient should receive (or have orders available for) an opiate or benzodiazepine or barbiturate infusion to reduce dyspnea, and the chart should document whether the patient has dyspnea. | 0 | - |
|  | IF a vulnerable elder who had dyspnea in the last 7 days of life died an expected death, THEN the chart should document how the dyspnea was treated and follow-up should be documented about the dyspnea. | 0 | - |
|  | IF a vulnerable elder who was conscious during the last 3 days of life died an expected death, THEN the medical record should contain documentation about pain or lack of pain during the last 3 days of life. | 0 | - |
|  | IF a vulnerable elder who was conscious during the last 3 days of life died an expected death, THEN the medical record should contain documentation about spirituality or how the patient was dealing with death or religious feelings. | 0 | - |
|  | IF a vulnerable elder without known family or next of kin died in the hospital, THEN the chart should document a search for next of kin. | 0 | - |
| **Gout** | IF a gout patient is receiving an initial prescription for allopurinol AND has significant renal impairment (defined as a serum creat ≥ 2 mg/dl or measured/estimated Cr Cl ≤ 50 ml/min) THEN the initial daily allopurinol dose should be less than 300 mg per day BECAUSE of the risk of allopurinol-related toxicity is increased in the presence of significant renal impairment in gout patients given a daily allopurinol dose equal to or exceeding 300 mg | 1 | 74 |
|  | Dose adjustment with concomitant use of allopurinol and azathioprine (see article) | 1 | 75 |
|  | Treatment of asymptomatic hyperuricaemia (see article) | 1 | 43 |
| **Hearing loss** | ALL vulnerable elders should have a hearing screening examination as part of the initial evaluation | 1 | 0 |
|  | IF a (vulnerable elder / person aged 65 or older -Steel-) (fails a hearing screening / has a problem with hearing -Steel-), THEN he or she should be offered a formal audiologic evaluation within 3 months | 2 | 85[85(76;94)] |
|  | IF a vulnerable elder has a hearing problem or fails an audiologic screening, THEN he or she should have an ear examination within 3 months. | 1 | 83 |
|  | IF a (vulnerable elder / person aged 65 or older -Steel-) is a hearing aid candidate, THEN he or she should be offered hearing rehabilitation | 2 | 67[67(50;83)] |
|  | If a vulnerable elder has hearing problem or fails an audiologic screening, then he or she should have an ear examination within 3 months | 0 | - |
|  | IF a vulnerable elder has conductive hearing loss, THEN he or she should be offered a referral to an otolaryngologist. | 0 | - |
| **Heart failure** | IF a (vulnerable elder / NH resident -Zingmond09-) has heart failure and left ventricular ejection fraction of 40% or less (or unknown) THEN he or she should be offered an ACE inhibitor or receptor blocker ^!^ | 4 | 57[65(33;87)] |
|  | Patient with heart failure, Prescribe ACEI or ARB; exclusions: ACEI, ARB exclusions | 1 | 48 |
|  | IF a vulnerable elder receives a new diagnosis of heart failure, THEN he or she should have a history taken at the time of diagnosis and hospitalization that documents the presence or absence of previous myocardial infarction, documented coronary artery disease, revascularization, current symptoms of chest pain or angina, history of hypertension, history of diabetes, history of hypercholesterolemia, history of valvular heart disease, history of thyroid disease, smoking, current medications, and a description of functional capacity (e.g., New York Heart Association functional status) | 1 | 83 |
|  | IF a vulnerable elder receives a new diagnosis of heart failure, THEN he or she should have the following elements of the physical examination documented at the time of presentation: weight, blood pressure, heart rate, lung examination, cardiac examination, and abdominal or lower-extremity examination | 1 | 100 |
|  | IF a (vulnerable elder / NH resident -Zingmond09-) receives a new diagnosis of heart failure, THEN (he or she should undergo the following studies within 1 month of the diagnosis (unless they have already been performed within the previous 3 months) -deleted Zingmond07-): chest radiography; electrocardiography; complete blood count; and (appropriate laboratory studies -Zingmond09-) measurement of serum sodium and potassium levels, serum creatinine concentration (electrolytes -Zingmond07-), and thyroid-stimulating hormone level ((in patients with atrial fibrillation or heart failure with no obvious cause) -deleted Zingmond07). | 3 | 44[36(27;67)] |
|  | IF a vulnerable elder receives a new diagnosis of heart failure, THEN education about disease management should be provided and documented | 2 | 37[37(23;50)] |
|  | IF a (vulnerable elder / NH resident -Zingmond09-) receives a new diagnosis of heart failure, THEN he or she should be offered an evaluation of left ventricular ejection fraction (within 1 month -deleted Zingmond09-). | 4 | 40[41(2;77)] |
|  | IF a vulnerable elder is hospitalized with heart failure, THEN he or she should have serum electrolyte levels, creatinine concentration, and blood urea nitrogen levels measured within 1 day of hospitalization | 1 | 100 |
|  | IF a vulnerable elder has heart failure, left ventricular ejection fraction of 40% or less, and New York Heart Association class I to III disease, THEN he or she should be offered a blocker, unless a contraindication (e.g., uncompensated heart failure) has been documented | 1 | 48 |
|  | IF a vulnerable elder has heart failure, (has left ventricular ejection fraction of 40% or less -deleted Zingmond07-), and does not have atrial fibrillation, THEN (from among the 3 generations of calcium-channel blocker medications -deleted Zingmond07-), he or she should not be treated with a first- or second-generation calcium-channel blocker^!^ | 2 | 99[99(97;100)] |
|  | IF a vulnerable elder has heart failure (and left ventricular ejection fraction of 40% or less -deleted Zingmond07-), THEN he or she should not be treated with a type I antiarrhythmic agent unless an implantable cardioverter defibrillator is in place | 2 | 100[100(99;100)] |
|  | IF a vulnerable elder has heart failure and atrial fibrillation, THEN he or she should be offered anticoagulation to achieve an INR or 2.0 to 3.0.^! †^ | 2 | 68[68(65;71)] |
|  | IF a vulnerable elder has heart failure and atrial fibrillation, AND he or she has documented contraindications to anticoagulation, THEN he or she should be offered aspirin^! †^ | 2 | 47[47(33;61)] |
|  | (Patient / NH resident -Zingmond09-) with heart failure, Prescribe a ß blocker; exclusions: ß blocker exclusions^‡^ | 3 | 19[19(13;25)] |
|  | NH resident post-hospitalization for HF should have follow-up visit and weight measured within 14 d after discharge. | 1 | 30 |
|  | NH resident with HF treated with digoxin should have a digoxin level checked if a medication that can alter levels is added. | 1 | 26 |
|  | Cr measured if on digoxin | 1 | 72 |
|  | BP measured | 1 | 89 |
|  | Electrolyte monitoring during ACE inhib Rx | 1 | 27 |
|  | Electrolyte monitoring during diuretic Rx | 1 | 45 |
|  | Visit within 4 weeks after discharge | 1 | 67 |
|  | Weight loss counseling | 1 | 7 |
|  | Water weight management plan | 1 | 4 |
|  | Goal Setting | 1 | 4 |
|  | Diet counseling | 1 | 11 |
|  | LDL < 100 if CAD | 1 | 40 |
|  | BP<130/80 mm Hg post MI or LVEF < 40 | 1 | 65 |
|  | BP<140/90 mm Hg no MI and LVDF >40 | 1 | 58 |
|  | IF a vulnerable elder with heart failure is treated with digoxin, THEN the digoxin level should be checked within 1 week if signs of toxicity develop. | 0 | - |
| **Hospital care** | IF a vulnerable elder is admitted to the hospital for any acute or chronic illness or any surgical procedure, THEN the evaluation should include, within 24 hours, 1) diagnoses and 2) prehospital and current medications | 1 | 97 |
|  | IF a vulnerable elder is admitted to the hospital for any acute or chronic illness or any surgical procedure, THEN documentation of cognitive status should be performed within 24 hours | 2 | 13[13(5;20)] |
|  | IF a vulnerable elder enters the hospital, THEN discharge planning should begin within 48 hours | 2 | 68[68(68,4)] |
|  | IF a vulnerable elder has valvular or congenital heart disease, intracardiac valvular prosthesis, hypertrophic cardiomyopathy, mitral valve prolapse with regurgitation, or previous episode of endocarditis, and a high-risk procedure is planned, THEN endocarditis prophylaxis should be given. | 1 | 100 |
|  | IF a hospitalized vulnerable elder is at very high risk for venous thrombosis, THEN the patient should have venous thromboembolism prophylaxis | 2 | 91[91(81;100)] |
|  | IF a hospitalized vulnerable elder has risk factors for stress peptic ulcers, THEN the patient should receive prophylaxis with an H2-blocker, sucralfate, or a proton-pump inhibitor.^!^ | 1 | 45 |
|  | IF a hospitalized vulnerable elder has a definite or suspected diagnosis of delirium, THEN an evaluation for potentially precipitating factors must be undertaken and identified causes treated. | 2 | 60 |
|  | IF a hospitalized vulnerable elder has a definite or suspected diagnosis of delirium, THEN identified potential causes should be treated | 1 | 44 |
|  | NH resident hospitalized for nonemergent revascularization or aneurism repair should have had cardiac stress test within 12 mos of operation. | 1 | 12 |
|  | If a vulnerable elder is admitted to a hospital or is new to a physician practice, then assessment of functional status should be documented | 1 | 95 |
|  | IF a vulnerable elder with heart failure is treated with digoxin, THEN the digoxin level should be checked within 1 week if signs of toxicity develop. | 0 | - |
|  | IF a vulnerable elder enters the hospital for nonemergent peripheral revascularization or aortic abdominal aneurysm repair, THEN a cardiac stress test should be performed if one was not performed in the previous year. | 0 | - |
|  | IF a hospitalized vulnerable elder has a new fever (body temperature, 38.5 °C [101.3 °F]), THEN there should be documentation that a physician examination was performed within 4 hours (or fever evaluation performed in the last 48 hours or an alternative explanation for the fever documented in the chart). | 0 | - |
|  | IF a hospitalized vulnerable elder has a definite or suspected diagnosis of delirium, THEN an evaluation for potentially precipitating factors must be undertaken and identified causes treated. | 0 | - |
| **Hypertension** | 1 IF a (vulnerable elder / NH resident -Zingmond09-) has newly diagnosed hypertension, THEN electrocardiography (and appropriate laboratory studies -Zingmond09-) (should be performed within 4 weeks of the diagnosis -deleted Zingmond09-) | 3 | 32[33(28;36)] |
|  | IF a vulnerable elder has a new diagnosis of hypertension, THEN there should be documentation regarding the presence or absence of other cardiovascular risk factors | 1 | 33 |
|  | IF a vulnerable elder receives a diagnosis of hypertension and the blood pressure is below 170/90 mm Hg, THEN there should be evidence that 3 or more blood pressure measurements of 140/90 mm Hg or greater were obtained before the diagnosis | 1 | 33 |
|  | IF a vulnerable elder receives a diagnosis of hypertension, THEN nonpharmacologic therapy with lifestyle modification for treatment of hypertension should be recommended, including dietary sodium restriction and weight loss if the patient is more than 10% over ideal body weight | 1 | 33 |
|  | IF a (vulnerable elder / person aged 50 or older -Steel-) remains hypertensive after nonpharmacologic intervention, THEN pharmacologic antihypertensive treatment should be initiated^!^ | 1 | 68[68(64;73)] |
|  | IF a vulnerable elder requires pharmacotherapy for treatment of hypertension in the outpatient setting, THEN a once- or twice-daily medication should be used unless there is documentation regarding the need for agents that require more frequent dosing^!^ | 1 | 93 |
|  | IF a (vulnerable elder / NH resident -Zingmond09-) has hypertension and has (renal parenchymal disease with a serum creatinine concentration greater than 1.5 mg/dL or more than 1 g of protein/24 h of collected urine / renal insufficiency or proteinuria -Zingmond07- / renal disease -Zingmond09-), THEN therapy with an ACE inhibitor should be (offered / prescribe -Zingmond07-)(ACE/ARB exclusions -Zingmond07-)^!^ | 3 | 45[39(31;63)] |
|  | IF a (vulnerable elder / NH resident -Zingmond09-) has hypertension and asthma, THEN blocker therapy for hypertension should not be used^!^ | 3 | 88[86(78;100)] |
|  | NH resident newly prescribed a diuretic should have electrolytes checked in 10 d | 1 | 11 |
| **Ischemic heart disease** | IF a (vulnerable elder / NH resident -Zingmond09-) is hospitalized with acute myocardial infarction, THEN he or she should be offered assessment of left ventricular function ((before discharge or -deleted Zingmond07-) within 3 days after hospital discharge -deleted Zingmond09-) | 3 | 61[63(50;69)] |
|  | IF a vulnerable elder has an acute myocardial infarction or unstable angina, (did not undergo angiography, and does not have contraindications to revascularization, THEN he or she should be offered noninvasive -deleted Zingmond07-)(THEN perform -Zingmond07-) stress testing 4 to 21 days after the infarction or anginal event. | 2 | 10[10(0;19)] |
|  | IF a vulnerable elder has an acute myocardial infarction or unstable angina, THEN he or she should be given aspirin therapy within 1 hour of presentation | 1 | 0 |
|  | IF a vulnerable elder has unstable angina or an acute myocardial infarction, THEN he or she should be offered blocker therapy within 12 hours of presentation | 1 | 50 |
|  | IF a vulnerable elder has an acute myocardial infarction that is measurable by electrocardiography and does not have contraindications to reperfusion therapy, THEN he or she should be offered treatment with reperfusion therapy | 1 | [*] |
|  | IF a vulnerable elder without contraindications to revascularization has an acute myocardial infarction(Zingmond2007) or unstable angina with 1 or more of the following—pain refractory to medical therapy (1 h of aggressive medical therapy), recurrent angina or ischemia at rest or with low-level activities, ischemia accompanied by symptoms of heart failure—THEN he or she should be offered urgent catheterization | 1 | 31 |
|  | IF a vulnerable elder has significant left main or significant 3-vessel coronary artery disease with left ventricular ejection fraction less than 50%, THEN he or she should be offered coronary artery bypass graft surgery | 1 | 0 |
|  | IF a vulnerable elder has established coronary artery disease and his or her cholesterol level (lipids -Zingmond07-) is not known, THEN (check lipids -Zingmond07-) he or she should undergo a fasting cholesterol evaluation, including total LDL and HDL cholesterol levels | 3 | 39[33(31;52)] |
|  | IF a (vulnerable elder / NH resident -Zingmond09-) has (established CHD and an LDL cholesterol level greater than 130 mg/dL / hypercholesterolemia -Zingmond07/Zingmond09-), (and a trial of step II diet therapy was not offered or was ineffective -deleted Zingmond07/Zingmond09-), THEN he or she should be (offered / prescribe -Zingmond07/Zingmond09-) cholesterol-lowering medication^!^ | 4 | 48[47(28;64)] |
|  | IF a vulnerable elder has established CHD and is not taking warfarin, THEN he or she should be offered antiplatelet therapy^!^ | 2 | 68[66(58;66)] |
|  | IF a vulnerable elder with established CHD smokes, THEN he or she should be offered counseling for smoking cessation at least annually and have this documented in the medical record | 1 | 50 |
|  | IF a vulnerable elder has had a recent myocardial infarction or recent coronary bypass graft surgery, THEN he or she should be offered cardiac rehabilitation | 1 | 0 |
|  | IF a (vulnerable elder / NH resident -Zingmond09-) has had a myocardial infarction, THEN he or she should be (offered a blocker / prescribe ß blocker -Zingmond07/Zingmond09-;) (exclusions: ß-blocker exclusions -Zingmond07-)^!^ | 3 | 47[38(21;53)] |
| **Malnutrition** | ALL vulnerable elders should be weighed at each physician office visit, and these weights should be documented in the medical record. | 1 | 42 |
|  | IF a vulnerable elder has involuntary weight loss of more than 10% of body weight over 1 year or less, THEN weight loss (or a related disorder) should be documented in the medical record as an indication that the physician recognized malnutrition as a potential problem. | 1 | 77 |
|  | IF a vulnerable elder has documented involuntary weight loss or hypoalbuminemia (_3.5 g/dL), THEN she or he should receive an evaluation for potentially reversible causes of poor nutritional intake. | 1 | 52 |
|  | IF a vulnerable elder has documented involuntary weight loss or hypoalbuminemia (_3.5 g/dL, THEN he or she should receive an evaluation for potentially relevant comorbid conditions, including medications that might be associated with decreased appetite (e.g., digoxin, fluoxetine, anticholinergics), depressive symptoms, and cognitive impairment | 1 | 76 |
|  | IF a vulnerable elder is hospitalized, THEN his or her nutritional status should be documented during the hospitalization by evaluation of oral intake or serum biochemical testing (e.g., albumin, prealbumin, or cholesterol). | 2 | 47 |
|  | IF a (vulnerable elder / NH resident -Zingmond09-) who was hospitalized for a hip fracture has evidence of nutritional deficiency ((thin body habitus or low serum albumin or prealbumin levels) -deleted Zingmond09-), THEN (oral or enteral nutritional protein–energy supplementation should be initiated postoperatively / should receive protein-energy supplementation -Zingmond09-). | 2 | 54 |
|  | IF a stroke patient has persistent dysphagia at 14 days, THEN a gastrostomy or jejunostomy tube should be considered for enteral feeding. | 1 | [*] |
|  | NH resident with a newly placed feeding tube should first have received a nutrition consult, feeding aid, or supplements. | 1 | 83 |
|  | IF a hospitalized vulnerable elder is unable to take foods orally for more than 72 hours, THEN alternative alimentation (for example, enteral or parenteral) should be offered. | 0 | - |
| **Medication management** | IF a vulnerable elder is prescribed a new drug, THEN the prescribed drug should have a clearly defined indication documented in the record^!^ | 1 | 98 |
|  | IF a vulnerable elder is prescribed a new drug, THEN the patient (or, if incapable, a caregiver) should receive education about the purpose of the drug, how to take it, and expected sides effects or important adverse reactions^! †^ | 2 | 18[18(17;18)] |
|  | EVERY new drug that is prescribed to a vulnerable elder on an ongoing basis for a chronic medical condition should have a documentation of response to therapy within 6 months^! †^ | 1 | 65 |
|  | ALL vulnerable elders should have a drug regimen review at least annually^! †^ | 1 | 68 |
|  | IF a (vulnerable elder / NH resident -Zingmond09-) is prescribed warfarin, THEN an INR should be determined within 4 days after initiation of therapy and at least every 6 weeks^!^ | 3 | 69[78(45;84)] |
|  | IF a vulnerable elder is prescribed warfarin, THEN an INR should be determined at least every 6 weeks^!^ | 1 | 53 |
|  | IF a vulnerable elder is prescribed a thiazide or loop diuretic, THEN he or she should have electrolytes checked at least yearly^!^ | 2 | 84[84(80;87)] |
|  | IF a (vulnerable elder / NH resident -Zingmond09-/ patient with diabetes -Zingmond07) (is prescribed an oral hypoglycemic drug -deleted Zingmond07-), THEN chlorpropamide should not be used^.!^ | 3 | 99[99(99;100)] |
|  | ALL (vulnerable elders / NH residents -Zingmond09-) should not be prescribed a medication with strong anticholinergic effects if alternatives are available^!^ | 3 | 88[84(82;98)] |
|  | IF a (vulnerable elder / NH resident -Zingmond09-) does not need control of seizures, THEN barbiturates should not be used^!^ | 3 | 98[99(96,6;99)] |
|  | IF a (vulnerable elder / NH resident -Zingmond09-/cadogan) (requires analgesia / All patients -Zingmond07-), THEN meperidine should not be used^!^ | 4 | 99[99(99;100)] |
|  | IF a vulnerable elder is newly started on a diuretic, THEN serum potassium and creatinine levels should be checked within 1 month of the initiation of therapy and then annually thereafter^!^ | 2 | 61[61(34;87)] |
|  | NH resident receiving a diuretic should have potassium measured annually | 1 | 87 |
|  | IF a vulnerable elder is (newly started on / prescribed -Zingmond07-) an ACE inhibitor (or ARB -Zingmond07-), THEN serum potassium and creatinine levels should be checked within (1 month / 4 wk -Zingmond07-) of the initiation of therapy^!^ | 3 | 49[37(88;22)] |
|  | NH resident newly prescribed an ACEI, ARB, or diuretic should have potassium and creatinine checked within 30 d and annually thereafter. | 1 | 32 |
|  | (Patient / NH resident older than 75 -Zingmond09-) treated with warfarin, history of PUD or GI bleeding, AND treated with NSAIDS, Prescribe misoprostol or a proton pump inhibitor medication^‡^ | 3 | 25[25(24;27)] |
|  | For ALL vulnerable elders the outpatient medical record of every physician and the hospital medical record should contain an up-to-date medication list. | 0 | - |
| **Osteoarthritis** | IF a (vulnerable elder / person age 75 or older -Ganz-) receives a diagnosis of symptomatic osteoarthritis, THEN functional status and degree of pain should be assessed annually^! †^ | 2 | 50[50(40;61)] |
|  | IF a vulnerable elder has monoarticular joint pain associated with redness, warmth, or swelling and the patient also has an oral temperature greater than 38.0 °C and does not have a previously established diagnosis of pseudogout or gout, THEN a diagnostic aspiration of the painfully swollen red joint should be performed that day. | 1 | [*] |
|  | IF an ambulatory vulnerable elder (ambulatory NH resident Cadogan )receives a new diagnosis of symptomatic osteoarthritis of the knee and has no contraindication to exercise, and is physically and mentally able to exercise, THEN a directed or supervised strengthening or aerobic exercise program should be prescribed within 3/1 months of diagnosis | 2 | 31[31(16;46)] |
|  | IF an ambulatory (vulnerable elder / person age 75 or older -Ganz-) has had a diagnosis of symptomatic osteoarthritis of the knee for more than (12 / 3 -Ganz-) months, has no contraindication to exercise, and is physically and mentally able to exercise, THEN there should be evidence that a directed or supervised strengthening or aerobic exercise program was prescribed at least once since the time of diagnosis | 2 | 22[22(0;44)] |
|  | IF an ambulatory (vulnerable elder / person age 75 or older -Ganz-) has had a diagnosis of symptomatic osteroarthritis for more than 6 months, THEN there should be evidence that education regarding the natural history, treatment, and self-management of the disease was offered at least once | 2 | 52,[52(36;69)] |
|  | IF an ambulatory vulnerable elder has had a diagnosis of symptomatic osteoarthritis of the knee for more than 12 months, THEN there should be evidence that the patient was offered education at least once since the time of diagnosis | 1 | 33 |
|  | IF oral pharmacologic therapy is initiated to treat osteoarthritis, THEN acetaminophen should be the first drug used, unless there is a documented contraindication to use ^! †^ | 3 | 53[43(26;59)] |
|  | IF oral pharmacologic therapy for osteoarthritis is changed from acetaminophen to a different oral agent, THEN there should be evidence that the patient has had a trial of maximum-dose acetaminophen (suitable for age and comorbid conditions). | 2 | 35[33(33,37)] |
|  | IF a (vulnerable elder / person age 75 or older -Ganz-) is treated with cyclooxygenase nonselective NSAIDS, THEN there should be evidence that the patient was advised of the risks associated with these drugs | 2 | 21[21(4;39)] |
|  | IF a person age 75 or older is treated with a COX-2 NSAID, THEN the patient should be advised of the risks associated with the drug. | 1 | 50 |
|  | IF a vulnerable elder (NH resident Cadogan) is older than age 75 years or has a history of peptic ulcer disease, gastrointestinal bleeding, or current coumadin use, AND the patient is being treated with a cyclooxygenase nonselective NSAID, THEN he or she should be offered concomitant treatment with misoprostol or a proton-pump inhibitor! | 2 | 11 |
|  | IF a person age 75 or older is treated with a COX nonselective NSAID, THEN he or she should be offered concomitant treatment with either misoprostol or a proton-pump inhibitor. | 1 | 27,4 |
|  | IF a (vulnerable elder / person age 75 or older -Ganz- / a person aged 50 or older -Steel-) with severe symptomatic osteoarthritis of the knee or hip has not responded to nonpharmacologic and pharmacologic therapy, THEN the patient should be offered referral to an orthopedic surgeon to be evaluated for total joint replacement within 6 months unless a contraindication to surgery is documented | 2 | 66[73(90;36)] |
|  | NH resident is bedfast should receive mobilization | 1 | 30 |
| **Osteoporosis** | ALL female vulnerable elders should be counseled at least once regarding intake of dietary calcium and vitamin D and weight-bearing exercises | 1 | 47 |
|  | ALL female vulnerable elders who smoke should be counseled annually about smoking cessation. | 1 | 48 |
|  | ALL female vulnerable elders should be counseled about estrogen replacement therapy at least once | 1 | 23 |
|  | IF a vulnerable elder has a new diagnosis of osteoporosis, THEN during the initial evaluation period an underlying cause of osteoporosis should be sought by checking medication use and current alcohol use | 1 | 42 |
|  | IF an ambulatory vulnerable elder has an osteoporotic fracture diagnosed, THEN physical therapy or an exercise program should be offered within 3 months | 1 | 0 |
|  | IF a (vulnerable elder / person aged 50 or older -Steel-) has (untreated -Steel-) osteoporosis, THEN calcium and vitamin D (and biphosphonate-Spinewine-) supplements should be recommended at least once^! †^ | 2 | 27[27(26;28)] |
|  | IF a vulnerable elder is taking corticosteroids for more than 1 month, THEN the patient should be offered calcium and vitamin D. ^!^ | 2 | 65[62(54;71)] |
|  | IF a female (vulnerable elder / aged 50 or older -Steel- / NH resident -Zingmond09-) has a new diagnosis of osteoporosis, THEN the patient should be offered (pharmacologic treatment -Zingmond09- /treatment with hormone replacement therapy, bisphosphonates, a selective estrogen receptor modulator (or PTH -Zingmond07- / or calcium and vitamin D -Steel-) or calcitonin within 3 months of diagnosis)^!^ | 4 | 44[46(20;60)] |
|  | IF a male vulnerable elder has osteoporosis and is hypogonadal, THEN he should be offered testosterone treatment | 1 | [*] |
|  | IF an ambulatory NH resident is newly diagnosed with symptomatic osteoarthritis (OA) of the knee, has no contraindication to exercise, and is physically and mentally able to exercise, THEN a directed or supervised | 1 | 46 |
| **Pain management** | ALL (vulnerable elders / NH residents –Cadogan-) should be screened for chronic pain during the initial evaluation period (with documentation in the primary care provider’s note during the initial evaluation period and at least quarterly -Cadogan-)^! †^ | 3 | 55[70(15;80,2)] |
|  | ALL vulnerable elders should be screened for chronic pain every 2 years.^! †^ | 1 | 41 |
|  | IF a vulnerable elder has a newly reported chronic painful condition, THEN a targeted history should be performed within 1 month^! †^ | 1 | 40 |
|  | IF a vulnerable elder has a newly reported chronic painful condition, THEN a physical examination should be performed within 1 month | 1 | 58 |
|  | IF a (vulnerable elder / NH resident -Cadogan-) has been prescribed a cyclooxygenase nonselective NSAID for treatment of chronic pain, THEN the medical record should indicate whether he or she has a history of peptic ulcer disease and, if a history is present, justification of NSAID use should be documented^! †^ | 2 | 11[11(10,12)] |
|  | IF a (vulnerable elder / NH resident -Cadogan-) with chronic pain is treated with opioids, THEN he or she should be offered a bowel regimen, or the medical record should document the potential for constipation or explain why bowel treatment is not needed^! †^ | 2 | 32[32(0;64)] |
|  | IF a vulnerable elder has a newly reported chronic painful condition, THEN treatment should be offered | 2 | 82[82(78,86)] |
|  | IF a (vulnerable elder/NH residents -Cadogan-) is treated for a chronic painful condition THEN he or she should be assessed for a response within 6/3 months! | 2 | 55[55(44;66)] |
|  | IF a NH resident has pain on MDS screen or is diagnosed with chronic pain, THEN the resident should be evaluated for depression by a PCP within 1 month | 1 | 49 |
|  | IF a NH resident has a positive MDS screen for pain, THEN a quantitative pain assessment using a standard pain scale should be used (with its use not precluded but modified for cognitive impairment) | 1 | 90 |
|  | IF a (vulnerable elder / NH resident -Cadogan-) has a newly reported painful condition, THEN a targeted H & P should be done by the PCP and documented within 1 month^‡^ | 1 | 10 |
|  | IF a patient is treated with a COX nonselective NSAID, THEN there should be evidence that the patient was advised of the risk for gastrointestinal bleeding associated with these drugs^‡^ | 1 | 54 |
| **Pneumonia** | IF a vulnerable elder with no history of allergy to the pneumococcal vaccine is not known to have already received a pneumococcal vaccine or if the patient received it more than 5 years ago (if before age 65 years), THEN a pneumococcal vaccine should be offered | 1 | 29 |
|  | IF a vulnerable elder has no history of anaphylactic hypersensitivity to eggs or to other components of the influenza vaccine, THEN the patient should be offered an annual influenza vaccination | 1 | 66 |
|  | IF a smoker develops pneumonia, THEN the smoker should be advised to quit smoking | 1 | 33 |
|  | IF a vulnerable elder is admitted to the hospital with pneumonia, THEN antibiotics should be administered within 8 hours of hospital arrival^!^ | 1 | 88 |
|  | IF a vulnerable elder is admitted to the hospital with community-acquired pneumonia with hypoxia, THEN the patient should receive oxygen therapy | 1 | 100 |
|  | IF a vulnerable elder has an empyema, THEN drainage is required | 1 | [*] |
|  | IF a vulnerable elder with community-acquired pneumonia is to be discharged home, THEN the patient should not be unstable on the day before or the day of discharge | 1 | 100 |
|  | IF a vulnerable elder is hospitalized and he or she is eligible for vaccination (that is, is not up-to-date with and influenza (during flu season). | 0 | - |
|  | IF pneumococcal or influenza vaccination rates among patients of a health delivery organization are low (60% of persons at risk for pneumococcal and influenza disease and, 90% of institutionalized elderly), THEN methods to increase the rate of vaccination should be used. | 0 | - |
|  | IF a health care organization cares for vulnerable elders, THEN it should have a formal plan to offer and encourage influenza vaccination among its employees. | 0 | - |
|  | IF a vulnerable elder with community-acquired pneumonia is to be switched from parenteral to oral antimicrobial therapy, THEN the patient must meet all of the following criteria: a clinically improving condition, hemodynamic stability, and tolerance of oral medication or food and fluids. | 0 | - |
| **Pressure ulcer** | IF a (vulnerable elder / NH resident – Bates-Jansen - )is admitted to an intensive care unit or a medical–surgical unit of a hospital and is unable to reposition himself or herself or has limited ability to do so, THEN risk assessment for pressure ulcers should be performed on admission (and weekly for the first 4 weeks -Bates-Jansen-) | 3 | 74[62(59;100)] |
|  | IF a vulnerable elder is identified as at risk for pressure ulcer development or a pressure ulcer risk assessment score indicates that the person is at risk, THEN preventive intervention must be instituted within 12 hours, addressing repositioning needs and pressure reduction (or management of tissue loads). | 1 | 0 |
|  | IF a vulnerable elder is identified as at risk for pressure ulcer development and has malnutrition (involuntary weight loss of _10% of body weight over 1 year or low albumin or prealbumin levels), THEN nutritional intervention or dietary consultation should be instituted. | 1 | 83 |
|  | Is identified as at risk for Pus Then address 2-hour repositioning, pressure reduction, and nutritional status unless not needed or tolerates | 1 | 55 |
|  | Is identified as at risk for PU development, then implement pressure reduction | 1 | 88 |
|  | Is identified as at risk for PU development, Then implement repositioning every 2 hours | 1 | 31 |
|  | Has a PU, THEN assess nutritional status within 1 week | 1 | 80 |
|  | IF a (vulnerable elder / NH resident -Bates-Jansen-) presents with a pressure ulcer, THEN the pressure ulcer should be assessed for 1) location, 2) depth and stage, 3) size, and 4) presence of necrotic tissue. | 3 | 27[32(0;33)] |
|  | IF a vulnerable elder presents with a clean full-thickness pressure ulcer and has no improvement at 4 weeks post-treatment, THEN 1) the appropriateness of the treatment plan and 2) the presence of cellulitis or osteomyelitis should be assessed. | 1 | 50 |
|  | Has a full-thickness PU with no improvement in 4 weeks or a partial thickness PU with no improvement in 2 weeks, THEN reassess the treatment plan and Stage III/IV PU for cellulitis or osteomyelitis. | 1 | 17 |
|  | IF a vulnerable elder presents with a partial-thickness pressure ulcer and has no improvement at 2 weeks post-treatment, THEN the appropriateness of the treatment plan should be assessed. | 1 | 33 |
|  | IF a vulnerable elder presents with a full-thickness sacral or trochanteric pressure ulcer covered with necrotic debris or eschar, THEN debridement interventions using sharp, mechanical, enzymatic, or autolytic procedures should be instituted within 3 days of diagnosis. | 1 | 17 |
|  | IF a vulnerable elder with a full-thickness pressure ulcer presents with systemic signs and symptoms of infection such as elevated temperature, leukocytosis, or confusion and agitation, and these signs and symptoms are not due to another identified cause, THEN the ulcer should be debrided of necrotic tissue within 12 hours. | 2 | 0 |
|  | IF a vulnerable elder with a full-thickness pressure ulcer presents with systemic signs and symptoms of infection, such as elevated temperature, leukocytosis, or confusion and agitation, and these signs and symptoms are not due to another identified cause, THEN a tissue biopsy or needle aspiration sample should be obtained and sent for culture and sensitivity testing within 12 hours. | 2 | [*] |
|  | Has a (stage two or greater -Arora- )PU, THEN a topical antiseptic should not be used on the wound | 2 | 90[90(82;98)] |
|  | Has a clean full-thickness or a partial-thickness PU, Then a moist wound-healing environment should be provided with topical dressings | 2 | 59[59(40;78)] |
|  | IF a vulnerable elder has a stage 2 or greater pressure ulcer, THEN a topical antiseptic should not be used on the wound. | 0 | - |
| **Screening and prevention** | ALL vulnerable elders newly admitted to a physician practice should receive the elements of a comprehensive geriatric assessment within 6 months | 1 | 14 |
|  | ALL vulnerable elders newly admitted to a physician practice should receive within 6 months recommendations from the comprehensive geriatric assessment | 1 | 44 |
|  | IF the elements of a comprehensive geriatric assessment are performed, THEN follow up should assure the implementation of recommendations | 1 | 100 |
|  | ALL vulnerable elders should be screened to detect problem drinking and hazardous drinking by taking a history of alcohol use or by using standardized screening questionnaires (e.g., CAGE, AUDIT) at least once | 1 | 49 |
|  | ALL vulnerable elders should receive screening for tobacco use and nicotine dependence | 1 | 83 |
|  | IF a vulnerable elder uses tobacco regularly, THEN he or she should be offered counseling or pharmacologic therapy to stop tobacco use at least once | 3 | 65[56(38;83)] |
|  | ALL vulnerable elders should receive an assessment of their activity level and be provided with counseling to promote regular physical activity at least once | 2 | 43[43(12;74)] |
|  | ALL vulnerable elders should be offered screening for colorectal cancer at least once with fecal occult blood testing or should have had sigmoidoscopy in the past 5 years or colonoscopy in the past 10 years | 1 | 76 |
|  | IF a female vulnerable elder is younger than age 70 years, THEN she should be offered mammographic screening for breast cancer every 2 years | 1 | 100 |
| **Stroke and atrial fibrillation** | IF a male vulnerable elder has carotid artery symptoms, receives a diagnosis of TIA or nondisabling stroke, and has had carotid imaging documenting at least 70% carotid stenosis on the side ipsilateral to the hemisphere producing the symptoms, and the medical record does not document that no facility is available with 30-day morbidity and mortality rates of less than 6%, THEN he should receive referral for evaluation for carotid endarterectomy within 4 weeks of the diagnostic study or event, whichever is later | 1 | [*] |
|  | IF a male vulnerable elder has carotid artery symptoms and is diagnosed with TIA or nondisabling stroke, and the medical record does not document that the patient is not a candidate for carotid surgery, THEN a carotid artery imaging study should be performed within 4 weeks | 1 | 100 |
|  | IF a (vulnerable elder / NH resident -Zingmond09-) has atrial fibrillation (for more than 48 hours and -deleted Zingmond07/Zingmond09-) has any high-risk condition ((impaired left ventricular function; female _75 years of age; hypertension or systolic blood pressure _ 160 mm Hg; previous ischemic stroke, TIA, or systemic embolism) -deleted Zingmond07/Zingmond09-)), THEN (he or she should be offered oral anticoagulation, or antiplatelet therapy / prescribe anticoagulant -Zingmond07- ) (if the medical record documents a reason not to give anticoagulant therapy -deleted Zingmond09-) | 3 | 46[23(21;94)] |
|  | IF a (vulnerable elder / NH resident -Zingmond09-)) has a presumed stroke (with hemispheric symptoms -Zingmond09-), THEN CT or MRI of the head should be performed (before initiation or continuation of thrombolytic treatment, anticoagulant therapy, or antiplatelet therapy -deleted Zingmond09-) | 2 | 79[79(58;100)] |
|  | IF a vulnerable elder is taking warfarin for atrial fibrillation, THEN the INR should be checked within 4 days of the first dose and at least every 6 weeks. | 1 | 67 |
|  | IF a vulnerable elder is taking warfarin for atrial fibrillation, THEN the INR should be checked at least every 6 weeks. | 1 | 64 |
|  | IF a vulnerable elder has a diagnosis of acute atherothrombotic ischemic stroke or a TIA, THEN antiplatelet treatment should be offered within 48 hours following the stroke or TIA, unless the patient is already receiving anticoagulant treatment. | 1 | 100 |
|  | IF a vulnerable elder has a TIA or stroke, THEN the medical record should document that smoking status was assessed and that smokers were counseled to stop smoking. | 1 | 100 |
|  | IF a vulnerable elder is started on thrombolytic therapy for a stroke, THEN all of the following should be true: head CT or MRI should precede initiation of thrombolytic therapy; sulcal effacement, mass effect, edema, or possible hemorrhage should not be present on neuroimaging; time from symptom onset to initiation of thrombolytic therapy should be documented in the medical record and should not exceed 3 hours; absence of absolute contraindications to thromboylsis should be documented in the medical record; tissue plasminogen activator should be used; AND National Institute of Neurological Disorders and Stroke exclusion criteria should not be present. | 1 | [*] |
|  | IF a vulnerable elder is admitted to the hospital with a diagnosis of acute ischemic or hemorrhagic stroke, THEN he or she should be admitted to a specialized acute or combined acute and rehabilitative stroke unit, or hospital. transferred to a specialized stroke unit if such a unit is available in the hospital. | 1 | 50 |
|  | NH resident <70 yrs old has a thrombotic CVA or TIA and hypercholesterolemia should be offered treatment to lower cholesterol. | 1 | 31 |
|  | 3. IF for a vulnerable elder the combined risk of surgery (patient characteristics and hospital or surgeon experience) is 10% or greater, THEN carotid endarterectomy should not be performed. | 0 | - |
| **Urinary incontinence** | ALL vulnerable elders should have documentation of the presence or absence or urinary incontinence during the initial evaluation^! †^ | 1 | 50 |
|  | ALL vulnerable elders should annually have documentation of the presence or absence of urinary incontinence^! †^ | 1 | 31 |
|  | IF a (vulnerable elder / person aged 65 or older -Steel-) has new urinary incontinence that persist for more than 1 month(Gandadesigan) or urinary incontinence at the time of a new evaluation, THEN a targeted history should be obtained that documents each of the following: 1) characteristics of voiding, 2) ability to get to the toilet, 3) previous treatment for urinary incontinence, 4) importance of the problem to the patient, and 5) mental status.^!^ | 3 | 17[19(11;20)] |
|  | IF a (vulnerable elder / person aged 65 or older -Steel-) has new urinary incontinence(Gandadesigan) that persists for more than 1 month (or urinary incontinence at the time of a new evaluation -deleted Steel-), THEN a targeted physical examination should be performed that documents 1) a rectal examination and 2) a genital system examination (including a pelvic examination for women).^!^ | 3 | 34 [31(22;50)] |
|  | IF a (vulnerable elder / person aged 65 or older -Steel-) has new urinary incontinence (Gandadesigan) that persists for more than 1 month or urinary incontinence at the time of a new evaluation, THEN a dipstick urinalysis (and post-void residual / and/or midstream urine sample -Steel-) should be obtained.^!^ | 3 | 43[43(13;74)] |
|  | IF a (vulnerable elder / person aged 65 or older -Steel-) has new urinary incontinence or (or worsening -Gandadesigan-) urinary incontinence at the time of a new evaluation, THEN treatment options should be discussed.^!^ | 3 | 49[59(26;61)] |
|  | IF a (cognitively intact -deleted Zingmond09-) (vulnerable elder / NH resident -Zingmond09-) who is capable of independent toileting (has documented stress, urge, or mixed incontinence without evidence of hematuria or high post-void residual / with UI -Zingmond09-), THEN behavioral treatment should be offered^!^ | 3 | 22[13(5;49)] |
|  | IF a vulnerable elder undergoes surgery or periurethral injections for urinary incontinence, THEN subtracted cystometry should be performed before the procedure | 1 | 0 |
|  | IF a female vulnerable elder has documented stress urinary incontinence caused by isolated intrinsic sphincter deficiency or isolated intrinsic sphincter deficiency with coexistent hypermobility and she undergoes surgical correction, THEN a sling or artificial sphincter procedure should be used. | 1 | 100 |
|  | IF a vulnerable elder has clinically significant, newly discovered overflow urinary incontinence, and indwelling urethral catheterization is used, THEN there should be documentation that the patient is not a candidate for alternative interventions as a result of severe physical or mental impairments or does not want alternative interventions | 1 | [*] |
|  | Follow up on incontinence | 1 | 17 |
|  | Classify type of incontinence in medical record | 1 | 26 |
|  | NH resident with UI should be on a toileting assistance program | 1 | 98 |
| **Vision care** | ALL (vulnerable elders / NH resident -Zingmond09-) should be offered an eye evaluation (every 2 years / annually -Zingmond09-) (that includes the essential components of a comprehensive eye examination -deleted Zingmond07-) | 3 | 69[69(49;86)] |
|  | IF a vulnerable elder has sudden-onset visual changes, eye pain, corneal opacity, or severe purulent discharge, THEN the patient should be examined within 72 hours by an ophthalmologist. | 1 | 80 |
|  | IF a vulnerable elder develops progression of a chronic visual deficit that now interferes with his or her ability to carry out needed or desired activities, THEN he or she should have an ophthalmic examination by a person skilled at ophthalmic examination within 2 months. | 1 | 100 |
|  | IF a vulnerable elder is diagnosed with a cataract, THEN assessment of visual function with respect to his or her ability to carry out needed or desired activities should be performed every 12 months. | 1 | 31 |
|  | IF a vulnerable elder(NH resident) has a new diagnosis of primary open-angle glaucoma, THEN the initial evaluation of each eye should include the essential components of a comprehensive eye examination AND documentation of the optic nerve appearance, visual field testing, and determination of an initial target pressure. | 1 | [*] |
|  | NH resident with new primary open angle glaucoma should have a comprehensive eye exam | 1 | 41[41(32;50)] |
|  | NH resident with primary open angle glaucoma should have appropriate yearly eye exam | 1 | 27 |
|  | IF a vulnerable elder with diabetes has a retinal examination, THEN the presence and degree of diabetic retinopathy should be documented. | 1 | 88 |
|  | IF a (vulnerable elder / NH resident -Zingmond09-) receives a diagnosis of proliferative diabetic retinopathy, THEN a dilated eye examination should be performed at least every 4 months. | 3 | 22 |
|  | IF a vulnerable elder with diabetes receives a diagnosis of macular edema, THEN a dilated eye examination should be performed at least every 6 months. | 2 | 69[69(39;100)] |
|  | IF a (vulnerable elder / person aged 50 or older -Steel-) receives a diagnosis of a cataract that limits the patient’s ability to carry out needed or desired activities, THEN cataract extraction should be offered. | 2 | 72[76(57;86)] |
|  | IF a (vulnerable elder / NH resident -Zingmond09-) undergoes cataract surgery, THEN (a follow-up ocular examination should occur within 48 hours and -deleted Zingmond07/Zingmond09-) reexamination should occur within 3 months. | 3 | 43[16(14;100)] |
|  | IF a vulnerable elder with glaucoma experiences progressive optic nerve damage on visual field tests or optic nerve examination, THEN treatment should be reassessed or advanced at least every 3 months until the intraocular pressure is lowered by at least 20% or there is documentation that the vision loss has stabilized. | 1 | [*] |
|  | IF a vulnerable elder who has been prescribed an ocular therapeutic regiment becomes hospitalized, THEN the regimen should be administered in the hospital unless discontinued by an ophthalmologic consultant.^!^ | 1 | 83 |
|  | IF a vulnerable elder who uses corrective lenses for any activities of daily living is hospitalized (or in a nursing home) and his or her corrective lenses are at the hospital (or nursing home), THEN the corrective lenses should be readily accessible to the vulnerable elder. | 1 | 89 |
|  | IF a vulnerable elder with age-related macular degeneration has a new-onset change in vision, THEN he or she should have a dilated retinal examination of the affected eye within 3 days | 0 | - |
|  | IF a vulnerable elder with functional visual deficits has subjective improvement on refraction, THEN he or she should receive a primary or updated prescription for corrective lenses. | 0 | - |
| **Cardio-vascular disease** | IF a person aged 50 or older has had a previous stroke, THEN the patient should be offered antihypertensive medication. | 1 | 65 |
| **Patient-oriented questions** | Some people with diabetes receive training to help manage their diabetes themselves. Have you ever participated in a course or class about diabetes, or received special training on how you can live with your diabetes from day-today? | 1 | 25 |
|  | How much do you think you know about managing your diabetes? (‘just about everything you need to know’ or ‘most of what you need to know’) | 1 | 78 |
|  | Has a doctor or nurse explained high cholesterol in a way you could understand? | 1 | 78 |
|  | Have doctors or nurses taken your preferences into account when making treatment decisions about your high cholesterol? | 1 | 49 |
|  | Has a doctor or nurse explained high blood pressure in a way you could understand at any time since you were first told you had high blood pressure? | 1 | 68 |
|  | In general, have doctors or nurses given you any choice about how to treat your high blood pressure? | 1 | 42 |
|  | Has any doctor or nurse ever talked to you about what the specific purpose of the treatment for your arthritis or joint pain is? | 1 | 78 |

## Additional information per condition. QI: quality indicator.

| **Condition ID** | **Conditions** | **# of QI used** | **# of QIs in each cond. Above 50%** | **Percentage of QIs above 50%** | **Proportion(%) of QIs not used (from ACOVE1 set)** | **Mean of the Mean(per QI) per condition** |
| --- | --- | --- | --- | --- | --- | --- |
| 1 | Falls and morbidity disorder | 13 | 2 | 0,15 | 0 | 32,7 |
| 2 | Dementia | 15 | 6 | 0,40 | 28 | 46,1 |
| 3 | Continuity of care | 8 | 6 | 0,75 | 38 | 68,9 |
| 4 | Depression | 15 | 4 | 0,27 | 23 | 42,7 |
| 5 | Diabetes | 12 | 8 | 0,67 | 0 | 63,2 |
| 6 | End of life care | 9 | 4 | 0,89 | 35 | 54,3 |
| 7 | Gout | 3 | 2 | 0,67 | - | 64,0 |
| 8 | Hearing loss | 4 | 3 | 0,75 | 33 | 58,7 |
| 9 | Heart failure | 28 | 12 | 0,43 | 7 | 51,2 |
| 10 | Hospital care | 10 | 6 | 0,60 | 44 | 62,4 |
| 11 | Hypertension | 9 | 3 | 0,33 | 0 | 48,4 |
| 12 | Ischemic heart disease | 13 | 2 | 0,15 | 0 | 32,2 |
| 13 | Malnutrition | 8 | 5 | 0,63 | 12 | 61,6 |
| 14 | Medication management | 16 | 12 | 0,75 | 8 | 68,3 |
| 15 | Osteoarthritis | 14 | 3 | 0,21 | 0 | 36,3 |
| 16 | Osteoporosis | 10 | 1 | 0,10 | 0 | 37,6 |
| 17 | Pain management | 12 | 5 | 0,42 | 0 | 47,6 |
| 18 | Pneumonia | 7 | 4 | 0,57 | 36 | 69,3 |
| 19 | Pressure ulcer | 16 | 7 | 0,44 | 9 | 46,5 |
| 20 | Screening and prevention | 9 | 5 | 0,56 | 0 | 63,8 |
| 21 | Stroke and atrial fibrillation | 11 | 6 | 0,55 | 10 | 70,8 |
| 22 | Urinary incontinence | 13 | 2 | 0,15 | 0 | 40,6 |
| 23 | Vision care | 15 | 8 | 0,53 | 13 | 62,6 |
| 24 | Cardio vascular disease | 1 | 1 | 1,00 | - | 65,4 |
| 25 | Patient-oriented questions | 7 | 4 | 0,57 | - | 59,7 |
