# Supplementary material for: Assessing Quality of Care of Elderly Patients Using the ACOVE Quality Indicator Set: A Systematic Review
Source: PLoS One. 2011 Dec 16;6(12):e28631. doi: 10.1371/journal.pone.0028631 (PMC3241679; doi:10.1371/journal.pone.0028631)
Supplement: Table S2 — Measured mean pass rate of QIs per condition and proportion of unique (matched) QIs with mean score above 50% per condition. ‡: The pass rate is reported for both delirium and dementia. †: These QIs were about physical functioning. QI: quality indicator, VE: vulnerable elder(s), NH: Nursing home, PC: Primary care, PIM: Prescribing indicated Mediations, AIM: Avoiding Inappropriate Medication, ECD: Education, Continuity, and Documentation, MM: Medication Monitoring, GEM: Geriatric Evaluation and Management, CHF: Chronic Heart Failure, IHI BTS: Institute of Healthcare Improvement's Breakthrough Series, PC: Primary Care. *: The same patient population and dataset was used as in the Wenger et al. study [21], for these common QIs we only considered the pass rates reported in [21] for our analysis. (DOC) [file pone.0028631.s002.doc]

Table S2: Measured mean pass rate of QIs per condition and proportion of unique (matched) QIs with mean score above 50% per condition.

| **Conditions** | **Reference** | **Settings** | **Patient population** | **No. of QIs** | **Reported pass rate** | **% of QIs with mean score > 50% among unique QIs (No.) per condition** |
| --- | --- | --- | --- | --- | --- | --- |
| Continuity of care | Wenger et al.[21] (2003) | Two managed care organizations (US) | 372 community-dwelling VE | 8 | 80% | 75%(6/8) |
|  | Zingmond et al.[15] (2007) | PC (US) | Community-dwelling elderly >75 yrs | 1 | 76% |  |
| Dementia | Wenger et al.[21] (2003) | Two managed care organizations (US) | 372 community-dwelling VE | 9 | 35% | 40%(6/15) |
|  | Zingmond et al.[25] (2009 ) | NH (US) | 21657 NH residents | 2 | 9% |  |
|  | Zingmond et al.[15] (2007) | PC (US) | Community-dwelling elderly >75 yrs | 3 | 11% |  |
|  | Arora et al.[24] (2007) | Academic medical center (US) | 328 VE admitted at a general medicine ward‡ | 5 | 31% |  |
|  | Wenger et al.[26](2009) | Two managed care organizations (US) | Community dwelling elderly >75 yrs | 7 | 44% (in inter. group) vs 41% |  |
| Depression | Wenger et al.[21] (2003) | Two managed care organizations (US) | 372 community-dwelling VE | 13 | 31% | 26%(4/15) |
|  | Steel et al.[17] (2008) | Private households (UK)-PC | 8688 participants in the English longitudinal study of ageing | 3 | 64% |  |
|  | Zingmond et al.[25] (2009 ) | NH (US) | 21657 NH residents | 6 | 16% |  |
|  | Zingmond et al.[15] (2007) | PC (US) | Community-dwelling elderly >75 yrs | 5 | 33% |  |
| Diabetes mellitus | Wenger et al.[21] (2003) | Two managed care organizations (US) | 372 community-dwelling VE | 10 | 57% | 58%(7/12) |
|  | Steel et al.[17] (2008) | Private households (UK)-PC | 8688 participants in the English longitudinal study of ageing | 5 | 74% |  |
|  | Zingmond et al.[25] (2009 ) | NH (US) | 21657 NH residents | 3 | 49% |  |
|  | Zingmond et al.[15] (2007) | PC (US) | Community-dwelling elderly >75 yrs | 4 | 56% |  |
| End-of-life care | Wenger et al.[21] (2003) | Two managed care organizations (US) | 372 community-dwelling VE | 8 | 9% | 44%(4/9) |
|  | Zingmond et al.[25] (2009 ) | NH (US) | 21657 NH residents | 2 | 89% |  |
| Falls, instability and physical function | Rubenstein et al.*[12] (2004) | Two managed care organizations (US) (US) | 372 VE | 8 | 3 -71% | 15%(2 /13) |
|  | Wenger et al.[21] (2003) | Two managed care organizations (US) | 372 community-dwelling VE | 8 | 34% |  |
|  | Steel et al.[17] (2008) | Private households (UK)-PC | 8688 participants in the English longitudinal study of ageing | 2 | 44% |  |
|  | Zingmond et al.[25] (2009 ) | NH (US) | 21657 NH residents | 1 | 31% |  |
|  | Arora et al.[24] (2007) | Academic medical center (US) | 328 VE admitted at a general medicine ward † | 2 | 83% |  |
|  | Wenger et al.[26](2009) | Two community medical groups (US) | Community dwelling elderly >75 yrs (357 at intervention sites and 287 at control sites) | 5 | 44% (in inter. group) vs. 23% |  |
| Hearing loss | Wenger et al.[21] (2003) | Two managed care organizations (US) | 372 community-dwelling VE | 4 | 77% | 75%(3/4) |
|  | Steel et al.[17] (2008) | Private households (UK)-PC | 8688 participants in the English longitudinal study of ageing | 2 | 79% |  |
| Heart failure | Wenger et al.[21] (2003) | Two managed care organizations (US) | 372 community-dwelling VE | 12 | 71% | 46%(13/28) |
|  | Asch et al.[13] (2005) -) | 4 organizations participating in IHI BTS for CHF and 4 compatible comparison organizations (US) | 489 patients | 23 | Median pass rates for all the QIs increased from 57 (baseline)to 61%(after BTS) |  |
|  | Zingmond et al.[25] (2009 ) | NH (US) | 21657 NH residents | 6 | 23% |  |
|  | Zingmond et al.[15] (2007) | PC (US) | Community-dwelling elderly >75 yrs | 6 | 63% |  |
| Hospital care | Wenger et al.[21] (2003) | Two managed care organizations (US) | 372 community-dwelling VE | 8 | 61% | 60%(6/10) |
|  | Zingmond et al.[25] (2009 ) | NH (US) | 21657 NH residents | 1 | 12% |  |
|  | Arora et al.[24] (2007) | Academic medical center (US) | 328 VE admitted at a general medicine ward † | 4 | 82% |  |
| Hypertension | Wenger et al.[21] (2003) - | Two managed care organizations (US) | 372 community-dwelling VE | 8 | 77% | 33%(3/9) |
|  | Steel et al.[17] (2008) | Private households (UK)-PC | 8688 participants in the English longitudinal study of ageing | 1 | 72% |  |
|  | Zingmond et al.[25] (2009 ) | NH (US) | 21657 NH residents | 4 | 38% |  |
|  | Zingmond et al.[15] (2007) | PC (US) | Community-dwelling elderly >75 yrs | 3 | 51% |  |
| Ischemic heart disease | Wenger et al.[21] (2003) | Two managed care organizations (US) | 372 community-dwelling VE | 13 | 55% | 15%(2/13) |
|  | Steel et al.[17] (2008) | Private households (UK)-PC | 8688 participants in the English longitudinal study of ageing | 5 | 83% |  |
|  | Zingmond et al.[25] (2009 ) | NH (US) | 21657 NH residents | 3 | 22% |  |
|  | Zingmond et al.[15] (2007) | PC (US) | Community-dwelling elderly >75 yrs | 6 | 42% |  |
| Malnutrition | Wenger et al.[21] (2003) | Two managed care organizations (US) | 372 community-dwelling VE | 7 | 47% | 62%(5/8) |
|  | Zingmond et al.[25] (2009 ) | NH (US) | 21657 NH residents | 2 | 77% |  |
| Osteoporosis | Wenger et al.[21] (2003) | Two managed care organizations (US) | 372 community-dwelling VE | 9 | 36% | 21%(3/14) |
|  | Steel et al.[17] (2008) | Private households (UK)-pc | 8688 participants in the English longitudinal study of ageing | 2 | 53% |  |
|  | Zingmond et al.[25] (2009 ) | NH (US) | 21657 NH residents | 2 | 27% |  |
|  | Zingmond et al.[15] (2007) | PC (US) | Community-dwelling elderly >75 yrs | 1 | 39% |  |
| Osteoarthritis | Cadogan et al.[11] (2005) | 38 NHs (50-200 beds each) (US) | 542 NH residents | 3 | 26-46% | 10%(1/10) |
|  | Wenger et al.[21] (2003) | Two managed care organizations (US) | 372 community-dwelling VE | 11 | 31% |  |
|  | Ganz et al.[16] (2006) | Subgroup of 2 medical groups One primary care group and 1 special group (US) | 339 elderly arthritis patients (>75 yrs) | 8 | Overall QI pass rate: 57% pass rate range: 27 – 73% |  |
|  | Steel et al.[17] (2008) | Private households (UK) -PC | 8688 participants in the English longitudinal study of ageing | 4 | 29% |  |
|  | Zingmond et al.[25] (2009 ) | NH (US) | 21657 NH residents | 1 | 27% |  |
| Pain management | Cadogan et al.[11] (2005) | 38 NHs (50-200 beds each) (US) | 542 NH residents | 9 | 10-99%, | 58%(7/12) |
|  | Chodosh et al.*[22] (2004) | Two managed care plans (US) | 372 community-dwelling VE | 11 | 10 – 99% |  |
|  | Wenger et al.[21] (2003) | Two managed care organizations (US) | 372 community-dwelling VE | 8 | 51% |  |
|  | Steel et al.[17] (2008) | Private households (UK)-PC | 8688 participants in the English longitudinal study of ageing | 1 | 78% |  |
| Pressure Ulcer | Bates-Jensen et al.[18] (2003) | Eight NHs (US) | 191 NH residents | 9 | 0-98%. | 43% (7/16) |
|  | Wenger et al.[21] (2003) | Two managed care organizations (US) | 372 community-dwelling VE | 9 | 41% |  |
|  | Arora et al.[24] (2007) | Academic medical center (US) | 328 VEs admitted at a general medicine ward † | 5 | 76% |  |
| Pneumonia | Wenger et al.[21] (2003) | Two managed care organizations (US) | 372 community-dwelling VE | 7 | 49% | 57% (4/7) |
| Screening and prevention | Wenger et al.[21] (2003) | Two managed care organizations (US) | 372 community-dwelling VE | 9 | 67% | 55% (5/9) |
| Smoking | Steel et al.[17] (2008) | Private households (UK)-PC | 8688 participants in the English longitudinal study of ageing | 1 | 74% | - |
| Stroke and atrial fibrillation | Wenger et al.[21] (2003) | Two managed care organizations (US) | 372 community-dwelling VE | 10 | 82% | 54% (6/11) |
|  | Steel et al.[17] (2008) | Private households (UK)-PC | 8688 participants in the English longitudinal study of ageing | 1 | 65% |  |
|  | Zingmond et al.[25] (2009 ) | NH (US) | 21657 NH residents | 3 | 20% |  |
|  | Zingmond et al.[15] (2007) | PC (US) | Community-dwelling elderly >75 yrs | 1 | 23% |  |
| Urinary Incontinence | Schnelle et al.[10] (2003) | 18 NHs, 50-200 beds each (US) | 426 incontinent residents | 9 | 0-98%. | 23%(3/13) |
|  | Gnanadesigan et al.*[20] (2004) | 2 managed care plans (US) | 372 community-dwelling VE | 7 | 13-59% |  |
|  | Wenger et al.[21] (2003) | Two managed care organizations (US) | 372 community-dwelling VE | 10 | 29% |  |
|  | Steel et al.[17] (2008) | Private households (UK)-PC | 8688 participants in the English longitudinal study of ageing | 4 | 51% |  |
|  | Zingmond et al.[25] (2009 ) | NH (US) | 21657 NH residents | 2 | 97% |  |
|  | Wenger et al.[26](2009) | Two community medical groups (US) | Community dwelling elderly >75 yrs (357 at intervention sites and 287 at control sites) | 6 | 37% (in intervention group) vs. 22% |  |
| Vision | Wenger et al.[21] (2003) | Two managed care organizations (US) | 372 community-dwelling VE | 13 | 79% | 53%(8/15) |
|  | Steel et al.[17] (2008) | Private households (UK)-PC | 8688 participants in the English longitudinal study of ageing | 1 | 58% |  |
|  | Zingmond et al.[25] (2009 ) | NHs in 19 California counties (US) | 21657 NH residents | 5 | 37% |  |
|  | Zingmond et al.[15] (2007) | PC (US) | Community-dwelling elderly >75 yrs | 5 | 44% |  |
| Medication management /use | Wenger et al.[21] (2003) | Two managed care organizations (US) | 372 community-dwelling VE | 13 | 81% | 75%(12/16) |
|  | Zingmond et al.[25] (2009 ) | NH (US) | 21657 NH residents | 7 | 90% |  |
|  | Zingmond et al.[15] (2007) | PC (US) | Community-dwelling elderly >75 yrs | 8 | 83% |  |
| Pharmacological care | Higashi et al.*[23] (2004) | Two managed care organizations (US) | 372 community-dwelling VE | 43 | Overall pass rate: 50%. Per domain: PIM ; 97% AIM; 81% for ECD; 64% MM | - |
|  | Spinewine et al.[14] (2007) | 27 Acute GEM unit (Belgium) | 203 hospitalized patients aged 70 > yrs | 7 | 7 QIs for 6 conditions focusing on underuse: Osteoporosis (28%), Atrial fibrillation (61%), Ischemic heart disease (58%), Diabetes mellitus (60%), Heart failure (58%, 31%), and Myocardial infarction (39%). |  |
|  | Mikuls et al.[19] (2005) | All gout patients in an General Practice (US) | 63105 gout patients | 3 | QI pass rates range for allopurinol use in treatment of gout and asymptomatic hyperuricaemia: 25 to 57% |  |

‡: The pass rate is reported for both delirium and dementia. †: These QIs were about physical functioning. QI: quality indicator, VE: vulnerable elder(s), NH: Nursing home, PC: Primary care, PIM: Prescribing indicated Mediations, AIM: Avoiding Inappropriate Medication, ECD: Education, Continuity, and Documentation, MM: Medication Monitoring, GEM: Geriatric Evaluation and Management, CHF: Chronic Heart Failure, IHI BTS: Institute of Healthcare Improvement’s Breakthrough Series, PC: Primary Care.

*: The same patient population and dataset was used as in the Wenger et al. study [21], for these common QIs we only considered the pass rates reported in [21] for our analysis
