# Supplementary material for: Assessing Quality of Care of Elderly Patients Using the ACOVE Quality Indicator Set: A Systematic Review
Source: PLoS One. 2011 Dec 16;6(12):e28631. doi: 10.1371/journal.pone.0028631 (PMC3241679; doi:10.1371/journal.pone.0028631)
Supplement: Appendix S1 — Table of extracted data. ACOVE: Assessing Care Of Vulnerable Elders; VE: Vulnerable Elder; NH: Nursing Home; QoC: Quality Of Care; QI: Quality Indicators; IHI BTS: Institute of Healthcare Improvement's Breakthrough Series; CHF: Chronic Heart Failure; GEM: Geriatric Evaluation and Management; MAI: Medication Appropriateness Index; DM; Diabetes Mellitus; PU: Pressure Ulcer; MDS: Minimum Data Set; CI: Cognitive Impairment; UI: Urinary Incontinence; PIM: Prescribing Indicated Medication; AIM: Avoiding Inappropriate Medication; ECD: Education, Continuity, and Documentation; MM: Medication Monitoring; QoL: Quality of Life; RA: Rheumatoid Arthritis; AF: Atrial Fibrillation; OA: Osteoarthritis; 1:# = Number of quality indicators used. (DOC) [file pone.0028631.s003.doc]

# Appendix S1: table of extracted data

| **Author(s)** | **Study population (n)** | **Study design/**  **Period/**  **Setting** | **Objectives** | **Results** | **Conclusions** | **# QIs used^1^** | **Conditions** | **Source of information** |
| --- | --- | --- | --- | --- | --- | --- | --- | --- |
| Schnelle et al.[10] (2003) | 426 incontinent residents | Descriptive  18 NHs,  50-200 beds each (US) | Demonstrate reliability and feasibility of a protocol to assess urinary incontinence in NHs | High inter-rater agreement on scoring the QIs which facilitates replication (kappa 0.75 to 1.0); All NHs failed to document assessment for scheduling toileting | 1 to 2 days training in protocol is believed to be sufficient. Retrieval of archived data is costly. Good inter-rater reliability has been achieved | 9 | Urinary Incontinence care | Medical records and interviews |
| Cadogan et al.[11] (2005) | 542 NH residents | Descriptive  38 NHs (50-200 beds each) (US) | Demonstrate reliability and feasibility of a protocol to assess pain management in NHs and assess pain management quality | Excellent inter-rater reliability (kappa 0.65-1.00 and percentage agreement was 0.8-1.0). Pass rates: 10-99% | QIs can be reliably scored. Targeting residents with self-reported pain maximizes efficiency of the scoring system | 12 | Pain management and osteoarthritis | Medical records and interviews |
| Rubenstein et al.[12] (2004) | 372 VEs | Retrospective observational cohort study(13-month)  2 senior health care plans each with more than 20,000 enrollees (US) | Investigate quality of care for falls and instability | Of the 372 VEs, 57 had documentation of 69 episodes of 2 or more falls or fall with injury. Double this frequency was reported at interview.  47% of medical records of fallers consisted of: history of fall circumstances, co-morbidity, medications and morbidity. 85% documented 2 or more of the 4 elements. Documented physical examination was less complete. Recommendations were given in only 26% of cases | Community physicians seem to under-detect falls and gait disorders. Detected falls often receive inadequate evaluation. | 8 | Falls and instability | Medical records (including administrative data) and patient interview by telephone. Administrative data |
| Asch et al.[13] (2005) | 489 patients | Retrospective quasi experimental study  4 organizations participating in IHI BTS for CHF and 4 compatible comparison organizations (US) | Compare differences in indicator performance between baseline and post intervention periods for participating and nonparticipating organizations to evaluate the effects of the IHI BTS on QoC for chronic heart failure (CHF) | Participating organizations showed greater improvement for 11 of 21 indicators.  All indicators combined: participating sites improved more than controls (17 % vs 1% p< .0001)  Reliability: kappa 0.64 -0.78 for QI assessment | Organizations that participated in a disease-targeted collaborative provider interaction significantly improved counseling and education rates for CHF patients | 23 | Congestive heart failure | Medical records |
| Spinewine et al.[14] (2007) | 203 patients aged >70 | RCT with patients as unit of randomization  27 Acute GEM unit (Belgium) | To evaluate the effect of pharmaceutical care provided in addition to acute GEM care on the appropriateness of prescribing. using a protocol | Intervention patients had improvement in MAI and ACOVE underuse criteria from admission to discharge (OR: 9.1, 95% CI 4.2-21.6) | Pharmaceutical care in context of acute GEM improved appropriate use of medicine during hospital stay and after discharge | 7 | 7 underuse criteria, focusing on osteoporosis/fracture, atrial fibrillation, ischemic heart disease, diabetes mellitus, heart failure, myocardial infarction | Medical records and interviews |
| **Author(s)** | **Study population (n)** | **Study design/**  **Period/**  **Setting** | **Objectives** | **Results** | **Conclusions** | **# QIs used^1^** | **Conditions** | **Source of information** |
| Zingmond et al.[15] (2007) | 100528 patients | Observational cohort study(2 year)  Community-dwelling dual enrollees in Medicare and Medicaid, living in 19 California Counties (US) | To assess the applicability of process of care measures developed as part of ACOVE that were adapted previously for use with administrative data. To measure the QoC in community –dwelling VE. | 43/230 QIs were captured, overall QI pass rate was 65% (100528 patients with 930753 QIs, 9.3 QI/person) | The use of claims data-derived QoC process measures is feasible for the vulnerable older population but requires development of data elements focus on geriatric care | 43 | QIs that could be coded using administrative data by condition type or by intervention type from 22 conditions | Administrative data using linked Medicare and Medicaid |
| Ganz et al. [16] (2006) | 339 elderly (>75 yrs) arthritis patients | Observational cohort study  (13-months)  Subgroup of 2 medical groups. One primary care group and one specialty group (US). | To describe the quality of osteoarthritis care provided to community-dwelling elderly patients and to characterize arthritis-related function in these patients | Overall QI pass rate: 57.0% (CI95: 53.9-60.2). QI pass rates were higher for treatment QIs (63.5%, CI95: 59.8-67.2) than for medication safety QIs (43.8%, CI95: 38.2-49.4) | Quality of arthritis care for older adults is suboptimal, particularly with respect to medication safety.  Quality improvement efforts should target appropriate use of, and counselling regarding medications, as well as underuse of efficacious therapy. | 8 | Osteoarthritis care | Computer assisted telephone interview, Questionnaires |
| Steel et al. [17] (2008) | 8688 participants in the English longitudinal  study of ageing, of whom 4417 reported diagnoses of one or more of 13 conditions | Observational cohort study  Private households (UK) | To assess the receipt of effective healthcare  interventions in England by adults aged 50 or more with serious health conditions | Receipt of indicated care varied substantially by condition (29 to 83%). Substantially more indicated care was given for general medical conditions (74%, CI95: 73-76%) than for geriatric conditions (57%, CI95: 55-58%) | Shortfalls in QoC of adult >50 yrs with common health conditions in the UK were most noticeable in areas associated with disability and frailty | 32 | Stroke  Depression  DM, Falls  Hearing problems  Hypertension  Ischemic heart disease  Osteoarthritis  Osteoporosis  Pain management  Smoking cessation  Urinary incontinence  Vision | Patients (face to face interviews), expert panel |
| Bates-Jensen et al.[18] (2003) | 191 residents (elderly) | Observational study  Eight NHs (US) | Reliability and feasibility of a standardized protocol to score QIs for pressure ulcer care;  Assessment of Quality of PU care | Pass rate of the QIs ranged from 0-98%. Reliability medical record abstraction: kappa 0.689-1.00, agreement 80-100%; direct observation: kappa 0.979 and 0.928; thigh monitor: kappa 0.609-0.842 | Standardized QA system was feasible and had good reliability for 9 QIs for PU care | 9 | Pressure Ulcer Care | Medical records, direct observations, wireless thigh monitor observation data |
| Mikuls et al. [19] (2005) | 63105 gout patients | Retrospective database analysis (between 1990-1999)  All gout patients in a general practice research database (US) | To examine adherence to QIs concerning the quality of allopurinol use in the treatment of gout | 185 patients eligible for QI-1; 52 for QI-2 and 471 for QI-3. QI pass rates: 25 to 57%.  Male sex, older age, history of chronic renal failure and a greater number of concomitant drugs were significantly associated with increased odds of inappropriate treatment for asymptomatic hyperuricaemia. | Allopurinol is frequently prescribed inappropriately; future interventions should be aimed at high-risk groups incl. older men and those receiving multiple drugs | 3 | Allopurinol use in treatment of gout and asymptomatic hyperuricaemia | National medical record database |
| **Author(s)** | **Study population (n)** | **Study design/**  **Period/**  **Setting** | **Objectives** | **Results** | **Conclusions** | **# QIs used^1^** | **Conditions** | **Source of information** |
| Gnanadesigan et al.[20] (2004) | 372 community-dwelling VEs | Observational study, cohort 13-month  2 managed care plans (US) | To assess QoC provided to community-based VE with UI | In 32 patients with new or worsening UI, characteristics of voiding were documented for 75% of the patients; for 20% of the female patients pelvic examination was performed; for 42% of the male patients a rectal examination. Only 38% urinalysis, 16% a post void residual. Drug treatment was prescribed for 50% of the patients, Patient-behavioral treatments only for 13% | QoC for UI is inadequate, especially in primary care. Patient-behavioral treatments are rarely prescribed | 7 | Urinary Incontinence care | Medical record (explicit abstraction guidelines were used), telephone interview |
| Wenger et al.[21] (2003) | 372 community-dwelling vulnerable elderly | Observational cohort study, 13-month  2 managed care organizations (US) | To assess QoC provided to VE by evaluating the process of care using ACOVE QIs | Patients eligible for 10711 QIs, of which 55% were passed. No overall difference between the care organizations. Wide variation in QoC for condition, range 9-82% pass rate. Adherence to QIs concerning geriatric conditions lower than for general medical conditions (31% vs 52%, P<0.001). More treatment QIs were completed (81%) compared to the domains follow-up (63%), diagnosis (46%) and prevention (43%) | Care for VE is inadequate for a wide variety of conditions. The care for geriatric conditions is worse than for general medical conditions | 207 | 22 ACOVE conditions | Medical Record (185 QIs),  patient interview (22 QIs)  (approach: Written abstraction guidelines and real-time consultation with a senior nurse reviewer) |
| Chodosh et al.[22] (2004) | 372 community dwelling VEs (July 98-July 99) | Observational study  2 managed care plans (US) | Evaluation of QoC for chronic pain | <40% of VE were screened for pain in 2 years; 33% of VE had episode of pain during study period; 86% were offered treatment; 66% had follow-up; 10% of VE receiving NSAIDs received attention for GI toxicity; 61% of VE on opioids were offered laxatives | Chronic pain management in VE is inadequate. Improvement needed in screening, clinical evaluation, follow-up, attention to toxicities of therapy | 11 | Chronic Pain | Abstraction of administrative data & medical records on in- and outpatient, patient interviews. |
| Higashi et al.[23] (2004) | 372 community-dwelling VEs enrolled in managed care organizations  (July 98-July 99) | Observational cohort study.  2 managed care organizations (US) | Evaluation of QoC (Pharmacological Care) | Overall pass rate: 50% for PIM ; 97% AIM; 81% for ECD; 64% MM | Undertreatment, appropriate monitoring, documenting information, education of patients and maintaining continuity are more common problems than use of inappropriate medication | 43 | NA | Chart abstraction and patient interview |
| Arora et al. [24] (2007) | 328 VEs admitted at a general medicine ward | Prospective evaluation of QoC  Academic medical center (US) | Adaptation of ACOVE QIs for QoC assessment of hospitalized elderly and use of QIs for QoC measurement | Pass rate varied from 0-100%.  QIs general medical care higher pass rate than geriatric QIs (81,5% vs. 61,6% p<0.01) | Adherence to geriatric-specific QI’s lower than for general hospital care QIs. QI focusing on screening may overestimate performance | 16 | General medical  delirium and Dementia  Physical function  Pressure ulcer | Chart abstraction (computerized tool were used) and patient interview |
| **Author(s)** | **Study population (n)** | **Study design/**  **Period/**  **Setting** | **Objectives** | **Results** | **Conclusions** | **# QIs used^1^** | **Conditions** | **Source of information** |
| Zingmond et al.[25] (2009 ) | 21,657 NH registers, dually enrolled in Medicaid and Medicare. | Retrospective cohort study  Nursing homes in 19 California counties (US) | Assessing which clinical conditions are inadequately measured and adaption of ACOVE for use with routinely collected data | Only 50 of 283 QIs were captured. The overall QI pass rate was 76% QIs with highest pass rates measured avoidance of adverse medications and appropriate medication use | The use of claims data linked to MDS to measure the QoC is feasible for NH populations but assessment will be more valuable if additional data focused on geriatric care is used | 50 | 16 conditions:  Dementia, depression, diabetes, end-of-life care, falls, heart failure, hospital care, hypertension, ischemic heart disease, malnutrition, medication use, osteoarthritis, osteoporosis, stroke/AF, urinary incontinence, vision impairment | Administrative data |
| Wenger et al.[26] (2009) | Community dwelling elderly >75 yrs  (357 at intervention sites and 287 at control sites) | Controlled trial  2 community medical groups  (US) | To determine effect of a practice-based ACOVE-2 intervention on care for falls, UI and cognitive impairment | Intervention group patients received better care for falls (44% vs. 23%, p<0.001) and UI (37% vs. 22%, p<0.001), but not for cognitive impairment (44% vs. 41, p=.67) than control patients | The practice based intervention improved care for falls and UI, although quality remained low. More intensive interventions, such as embedding interventions components into an electronic medical record, are needed | 18 | Falls (5 QIs)  UI (6 QIs)  Dementia (7 QIs) | Medical records, interviews, administrative data |

ACOVE: Assessing Care Of Vulnerable Elders; VE: Vulnerable Elder; NH: Nursing Home; QoC: Quality Of Care; QI: Quality Indicators; IHI BTS: Institute of Healthcare Improvement’s Breakthrough Series; CHF: Chronic Heart Failure; GEM: Geriatric Evaluation and Management; MAI: Medication Appropriateness Index; DM; Diabetes Mellitus; PU: Pressure Ulcer; MDS: Minimum Data Set; CI: Cognitive Impairment; UI: Urinary Incontinence; PIM: Prescribing Indicated Medication; AIM: Avoiding Inappropriate Medication; ECD: Education, Continuity, and Documentation; MM: Medication Monitoring; QoL: Quality of Life; RA: Rheumatoid Arthritis; AF: Atrial Fibrillation; OA: Osteoarthritis;

1:# = Number of quality indicators used.
