# Supplementary material for: Assessing Quality of Care of Elderly Patients Using the ACOVE Quality Indicator Set: A Systematic Review
Source: PLoS One. 2011 Dec 16;6(12):e28631. doi: 10.1371/journal.pone.0028631 (PMC3241679; doi:10.1371/journal.pone.0028631)
Supplement: Diagram S1 — PRISMA Flow Diagram. (DOC) [file pone.0028631.s005.doc]

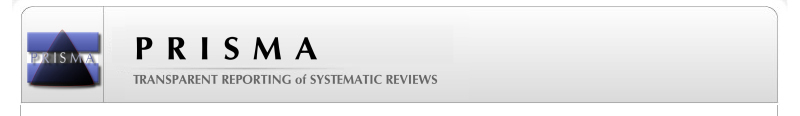
**PRISMA Flow Diagram**

**Screening**

**Included**

**Eligibility**

**Identification**

Records identified through database searching
(n = 429 )

Additional records identified through other sources
(n =0 )

Records after duplicates removed
(n =347 )

Records screened
(n = 347 )

Records excluded
(n = 302 )

Full-text articles assessed for eligibility
(n = 45 )

Full-text articles excluded, with reasons (n=28)

Not ACOVE related (n=6)

Main objective was not assessing the QoC (22)

Studies included in qualitative synthesis
(n =17 )

Studies included in quantitative synthesis (meta-analysis)
(n =0 )
